# Exploring the Antibacterial Potency of Cymbopogon Essential Oils: Liposome Encapsulation and Phytochemical Insights

**DOI:** 10.3390/antibiotics14050510

**Published:** 2025-05-15

**Authors:** Abdirahman Elmi, Fatouma M. Abdoul-Latif, Andréea Pasc, Arnaud Risler, Stéphanie Philippot, Ricardo Gil-Ortiz, Dominique Laurain-Mattar, Rosella Spina

**Affiliations:** 1 Centre d’Etudes et de Recherche de Djibouti, Medicinal Research Institute, IRM-CERD, Route de l’Aéroport, Haramous, Djibouti City B.P. 486, Djibouti; fatouma.abdoulatif@cerd.dj; 2 Université de Lorraine, CNRS, L2CM, F-54000 Nancy, France; andreea.pasc@univ-lorraine.fr (A.P.); arnaud.risler@univ-lorraine.fr (A.R.); stephanie.philippot@univ-lorraine.fr (S.P.); 3 Independent Researcher, E-46022 Valencia, Spain; rigilor@alumni.upv.es; 4 Université de Lorraine, INRAE, LAE, F-54000 Nancy, France; dominique.mattar@univ-lorraine.fr

**Keywords:** *Cymbopogon commutatus*, antibacterial activity, essential oil, liposome, encapsulation, gas chromatography

## Abstract

**Background**: Antimicrobial resistance (AMR) represents a critical global health challenge, requiring innovative strategies to combat resistant bacterial strains. *Cymbopogon* essential oils (EOs) are promising natural antimicrobial agents. **Methods**: The EO of *Cymbopogon commutatus* was extracted by hydrodistillation from fresh aerial parts and compared to commercial EOs from *C. citratus*, *C. nardus*, and *C. winterianus*. Antibacterial activity was evaluated against seven bacterial strains (two Gram-positive and five Gram-negative). Both water-soluble fractions and liposome-encapsulated formulations were tested. Liposomes were prepared using soybean lecithin, and their stability was assessed by dynamic light scattering (DLS). The chemical composition of the pure EOs, water-soluble fractions and non-water-soluble fractions was analyzed by gas chromatography–mass spectrometry (GC-MS). **Results**: Liposome encapsulation improved EO solubility in aqueous media and significantly enhanced antibacterial efficacy, reducing minimum inhibitory concentration (MIC) values compared to the water-soluble fractions (MICs ≥ 25%). Among the tested formulations, the liposome containing *C. citratus* EO exhibited the strongest inhibitory effect against *Staphylococcus aureus* (MIC: 0.04%) followed by liposomes with *C. nardus* and *C. commutatus* (MIC: 0.08%). Against *Enterococcus faecalis*, the most effective formulation was the liposome containing *C. winterianus* EO (MIC: 0.02%), followed by *C. citratus* (MIC: 0.08%). The liposome formulated with *C. winterianus* maintained its particle size over 72 h without phase separation. GC-MS analysis revealed distinct phytochemical profiles: *C. commutatus* EO was rich in piperitone (73.9%) and *C. citratus* was rich in (Z)-(3,3-Dimethyl)-cyclohexylideneacetaldehyde (39.9%) and citral (32.5%), while *C. nardus* and *C. winterianus* were dominated by geraniol (21.5%) and citronellal (30.8%), respectively. Notably, piperitone, the major compound in *C. commutatus* EO, exhibited strong antibacterial activity against *S. aureus* (MIC of <0.04%). **Conclusions**: These findings support the potential of liposome-encapsulated *Cymbopogon* EOs as an effective and sustainable strategy to address AMR. This study provides a foundation for the development of plant-based antimicrobial formulations with improved efficacy.

## 1. Introduction

Antimicrobial resistance (AMR) is a growing global health crisis, rendering existing antibiotics ineffective and contributing to severe infections, increased mortality, and escalating healthcare costs. Over 35,000 deaths annually in the U.S. are attributed to antibiotic-resistant infections [1]. This alarming trend highlights the urgent need for alternative therapeutic strategies to combat resistant bacterial strains.

Essential oils (EOs) are volatile, aromatic plant extracts rich in bioactive compounds, such as terpenes, phenols, alcohols, and aldehydes, known for their antimicrobial, antifungal, and antiviral properties. These natural products have been extensively studied for their potential in treating infections, particularly against multi-drug-resistant pathogens, such as methicillin-resistant *Staphylococcus aureus* (MRSA), vancomycin-resistant enterococci (VRE), and extended-spectrum β-lactamase (ESBL)-producing *Escherichia coli* [2]. For instance, EOs derived from *Melaleuca alternifolia* (tea tree), *Citrus aurantium* (bitter orange), and *Eucalyptus sp.* have demonstrated potent antibacterial activity [2,3,4].

Antimicrobial susceptibility testing (AST) assesses the effectiveness of antimicrobial agents against bacteria using standardized methods such as agar dilution, broth dilution, and disk diffusion [5]. These methods involve preparing bacterial suspensions to a specific density, exposing them to antimicrobial agents at defined concentrations, and incubating the samples under controlled conditions. The results help determine the susceptibility or resistance of bacterial strains to the tested agents, providing essential data for selecting appropriate antimicrobial therapies. The primary method used to determine the minimum inhibitory concentration (MIC) in antibacterial testing is the broth microdilution method. This method involves diluting the antibacterial agent in a liquid medium, typically using a 96-well microtiter plate. A bacterial sample is added to each well, and the MIC is identified as the lowest concentration at which no visible bacterial growth is observed after incubation.

Currently, there are no standardized regulations or recommendations for the in vitro evaluation of the antibacterial activity of essential oils (EOs) [5,6].

Despite their therapeutic potential, the application of EOs is hindered by their hydrophobic nature and limited solubility in aqueous media. Essential oils predominantly consist of low-polarity compounds that are only sparingly soluble in water but readily dissolve in organic solvents. This solubility challenge affects their efficacy in conventional antimicrobial assays and limits their bioavailability for clinical applications. Emulsions, micelles, or encapsulation in carriers like liposomes have been explored to overcome these limitations [3].

Encapsulation is a technique used to protect essential oils from volatilization, oxidation, and degradation, thereby improving their stability and enabling controlled release in various applications, including food preservation, pharmaceuticals, and cosmetics. Encapsulation protects essential oils from environmental factors such as light, oxygen, and heat, which can cause degradation. Studies have demonstrated that encapsulated essential oils retain their bioactive compounds more effectively over time, ensuring greater stability and prolonged efficacy. Several reviews have summarized the various techniques for the encapsulation of essential oils, highlighting advancements in the field [7,8,9,10].

Liposomes are spherical vesicles composed of phospholipid bilayers, and they represent particularly promising carriers for essential oils (EOs) in antimicrobial applications. Their bilayer structure closely mimics natural cell membranes, facilitating fusion or adhesion with bacterial or human membranes, which enhances the transport and cellular uptake of bioactive compounds [11,12].

This structural similarity is especially advantageous for overcoming bacterial resistance mechanisms, allowing active compounds to bypass or disrupt bacterial defenses more efficiently [7,13].

Encapsulation of EOs within liposomes offers multiple benefits. It improves the solubility and stability of these volatile compounds, protecting them from environmental degradation caused by light, heat, and oxygen. Furthermore, liposomes provide controlled and targeted release, maintaining therapeutic concentrations of active compounds over extended periods—an essential feature for sustained antimicrobial activity [12,13].

The liposomal formulation also enhances bioavailability and promotes a more effective penetration into bacterial cells, strengthening the antimicrobial efficacy of EOs [13].

In response to the inherent limitations of EO solubility in aqueous media, we replaced the aromatic water with an emulsion prepared using natural liposomes derived from soy lecithin, as the lipid source. This strategy significantly improved the dispersion of EOs in the aqueous phase, enabling a more accurate assessment of their antibacterial activity.

Overall, these properties make liposome-encapsulated essential oils a promising strategy for enhancing antimicrobial effectiveness, particularly against resistant bacterial strains.

**Figure 1:**
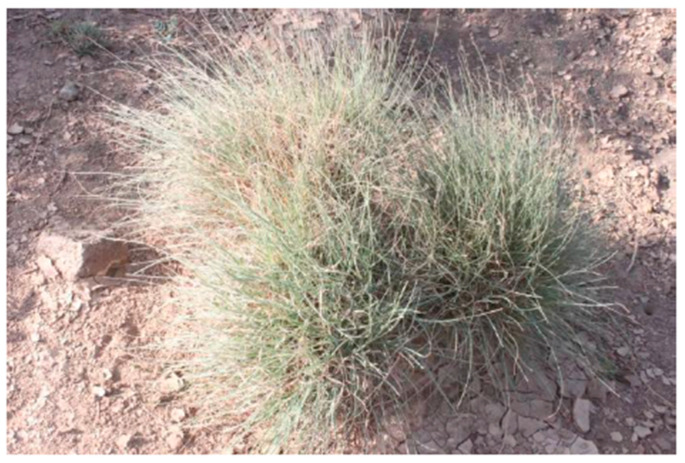
*C. commutatus* plant, collected from the locality of Omar-Jagga, Arta region (central Djibouti).

The genus *Cymbopogon*, which includes approximately 55 aromatic grass species, is well known for its EOs rich in monoterpenes and sesquiterpenes [14]. Common species such as *Cymbopogon citratus* (lemongrass), *C. winterianus* (Java citronella), and *C. nardus* (Ceylon citronella) have demonstrated antimicrobial activity in prior studies [15,16,17]. However, little is known about the EO of *Cymbopogon commutatus*, a species commonly found in Djibouti (Figure 1 and Figure S1).

This study aims to evaluate antibacterial activities and to characterize the chemical composition of *C. commutatus* EO from Djibouti, never described before, and the three commercially available *Cymbopogon* EOs (*C. citratus*, *C. nardus*, and *C. winterianus*). To address the solubility challenges of EOs in antibacterial assays, we encapsulated the EOs in natural soy lecithin-based liposomes and evaluated their stability, solubility, and antibacterial performance, highlighting their potential as sustainable solutions for combating AMR.

## 2. Results

### 2.1. Determination of Antibacterial Activity of Essential Oils (Eos) and EO-Liposome

The minimum inhibitory concentration (MIC) of the four essential oils (EOs) was evaluated against pathogenic bacterial strains. It is important to note that essential oils are immiscible in aqueous liquid media. Therefore, only the water-soluble fraction obtained from the different essential oils was used to perform the antibacterial activity tests, as described in the Section 4.

When evaluating the antibacterial activities of EOs, it is important to note that EOs do not fully solubilize in culture media, which are predominantly water-based. We followed the method described in the ISO 20776-1 standard [18], with some modifications (see Section 4 for details). Although this is the reference method for testing conventional antimicrobials, it is not a standard for assessing essential oils—no such standard currently exists. This approach was chosen, as in other studies, in order to follow a widely recognized and reproducible method.

In our preliminary experiments, different solubilizing agents, including DMSO, ethanol, and Tween 80, were evaluated for their ability to dissolve the essential oils. A concentration exceeding 20% of solvent or solubilizing agent was necessary to achieve effective solubilization. However, at such concentrations, some solvents, particularly DMSO and ethanol, displayed intrinsic antimicrobial activity against certain bacterial strains, potentially interfering with the assay results.

For this reason, we used liposomes prepared with soy lecithin, which alone did not exhibit any antimicrobial activity. This approach allowed us to avoid interference from solubilizing agents and to accurately assess the antimicrobial properties of the essential oil.

To perform the antimicrobial activity assays, the essential oils (EOs) were subjected to vigorous agitation with water using a vortex mixer. This process resulted in the formation of two distinct phases: a water-soluble fraction located beneath the EO layer and an upper phase containing the hydrophobic portion of the EO.

The hydrophilic EO compounds dissolved in the water, while the hydrophobic compounds remained in the upper phase. The water-soluble fraction, collected from the lower phase, was then used to conduct the antibacterial activity assays.

The water-soluble fractions of the four *Cymbopogon* species were tested against seven bacterial strains, including two Gram-positive bacteria (*Staphylococcus aureus* and *Enterococcus faecalis*) and five Gram-negative bacteria (*Pseudomonas aeruginosa*, *Escherichia coli ABC5*, *Klebsiella pneumoniae*, *Acinetobacter baumannii*, *Enterobactercloacae*). The results are visible in Table 1.

Minimum inhibitory concentrations (MICs) were expressed as % (*v*/*v*) relative to the samples tested against the target bacterial strains. For *C. citratus*, *C. commutatus*, *C. nardus*, and *C. winterianus*, MICs were determined from the water-soluble fractions. In contrast, for the formulations L + *C. citratus*, L + *C. commutatus*, L + *C. nardus*, and L + *C. winterianus*, MICs were assessed using pure essential oils incorporated into liposomes.

Among the tested essential oils, *C. commutatus* exhibited low activity, with an MIC of 25% against *S. aureus*, *E. faecalis*, *A. baumannii*, and *E. cloacae*. *C. citratus* also showed low activity, with an MIC of 25% against *S. aureus*, *A. baumannii*, and *E. coli*. *C. nardus* demonstrated the least antibacterial activity overall, with no significant inhibition (>25%) against most strains and only low activity against *E. cloacae* (MIC: 25%). *C. winterianus* exhibited low activity against *S. aureus*, *E. faecalis*, and *A. baumannii* (MIC = 25%), but was ineffective (>25%) against the remaining strains.

None of the essential oils were effective against *Pseudomonas aeruginosa* and *Klebsiella pneumoniae*, with MIC values exceeding 25% for all samples.

Gram-positive bacteria appeared more susceptible to the tested essential oils compared to Gram-negative bacteria. Among Gram-positive strains, *S. aureus* and *E. faecalis* were inhibited by *C. citratus*, *C. commutatus*, and *C. winterianus* at 25%. Among Gram-negative strains, activity was primarily observed against *A. baumannii* (25%) and *E. cloacae* (25%) with selected oils.

*C. commutatus* and *C. citratus* demonstrated broader antibacterial activity, particularly against Gram-positive strains and selectively against certain Gram-negative strains (*A. baumannii* and *E. cloacae*). In contrast, *C. nardus* showed limited activity.

To enhance the solubility of essential oil (EO) compounds in aqueous media and reassess their antibacterial activity, EOs were encapsulated in a simple liposome made of soy lecithin.

The antimicrobial activity of liposomes alone, the water-soluble fraction of the essential oil, and liposome-encapsulated essential oils (L + EOs) was evaluated against various bacterial strains, including both Gram-positive and Gram-negative species.

The encapsulation of *Cymbopogon* essential oils in liposomes significantly improved the solubilization of all their components and enhanced both their solubility and antibacterial efficacy (Table 1). The results demonstrate the potential of liposome-encapsulated essential oils, particularly *C. citratus* and *C. winterianus*, as effective antimicrobial agents against Gram-positive bacteria and selected Gram-negative strains.

The encapsulation of EOs significantly improved their antibacterial efficacy. Among the formulations, L + *Cymbopogon winterianus* (L + *C. wint.*) and L + *Cymbopogon citratus* (L + *C. citr.*) exhibited the strongest inhibitory effect against *Staphylococcus aureus*, with a minimum inhibitory concentration (MIC) of 0.02% and 0.04%, respectively. The L + *Cymbopogon nardus* (L + *C. nard.*) and L + *Cymbopogon commutatus* (L + *C. comm.*) showed moderate activity, with MICs of 0.08%. For *Enterococcus faecalis*, L + *Cymbopogon winterianus* (L + *C. wint.*) demonstrated the highest efficacy, achieving an MIC of 0.04%, followed by L + *C. citr.* at an MIC of 0.08%.

Regarding Gram-negative bacteria, L + *C. citr.* and L + *C. wint.* were effective against *Pseudomonas aeruginosa* and *Escherichia coli*, both with MICs of 0.08%, while L + *C. comm.* exhibited slightly reduced activity, with an MIC of 0.31%. The formulations were less effective against *Klebsiella pneumoniae*, yielding MIC values of >1.25% for L + *C. wint.* and >0.63% for L + *C. nard*. Against *Acinetobacter baumannii*, L + *C. nard.* showed the strongest inhibition, with an MIC of 0.04%, followed by L + *C. citr.* at 0.08% and L + *C. comm.* at 0.16% and L + *C. wint.* at >0.63%. For *Enterobacter cloacae*, L + *C. citr.* achieved an MIC of 0.08%, compared to L + *C. wint.*, which had an MIC of >0.63%.

These findings confirm that liposome encapsulation enhances both the solubility and antibacterial activity of *Cymbopogon* essential oils. Notably, the formulations L + *C. citratus* and L + *C. winterianus* exhibited the strongest antimicrobial effects against Gram-positive bacteria and selected Gram-negative strains.

Overall, the results demonstrate that combining liposomes with essential oils from *Cymbopogon* species consistently improves their antibacterial efficacy.

### 2.2. Determination of Stability of EO Microencapsulation

The stability of the emulsion of the natural liposome system composed of soy lecithin was also verified during biological tests, extending up to 72 h.

The evaluation of stability included both a visual inspection to assess homogeneity and the measurement of aggregate size using dynamic light scattering (DLS). DLS is a technique widely used to measure the size distribution of nanoparticles and submicron particles in suspension. In this method, a laser is directed at particles in a liquid medium, and the scattered light is analyzed to determine the size and distribution of the particles (Figures S2–S5).

**Figure 2:**
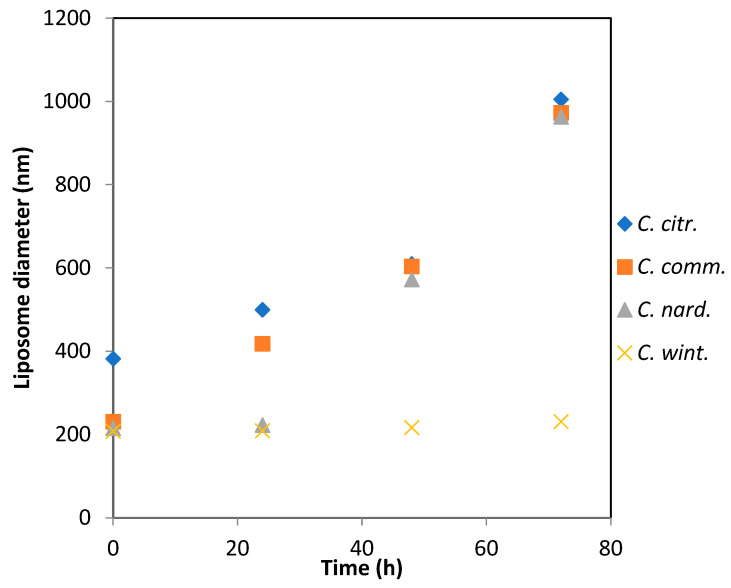
Emulsion stability of four liposomes containing *Cymbopogon* essential oils at the time of the bioassay, as measured by dynamic light scattering (DLS). (*C. citratus* is *C. citr.*, *C. commutatus* is *C. comm.*, *C. nardus* is *C. nard.* and *C. winterianus* is *C. wint.*).

No phase separation was observed up to 72 h. Liposomes with an approximate size of 200 nm were obtained via ultrasonication of an aqueous solution containing 0.3 wt% soy lecithin. The addition of *C. citratus* essential oil led to the formation of larger liposomes, with an average size of 380 nm (Figure 2). This increase in size is likely due to the encapsulation of the oil within the lipid bilayer.

The aggregate size of the emulsions prepared with *C. nardus* and *C. winterianus* remained stable over 24 h, in contrast to those prepared with *C. commutatus* and *C. citratus*. Beyond this period, only *C. winterianus* maintained a consistent average diameter, while the other three oils showed a linear increase in aggregate size, reaching approximately 1 μm at 72 h.

Figure 2 illustrates the changes in liposome diameter over time for the four tested samples: *C. citratus*, *C. commutatus*, *C. nardus*, and *C. winterianus*. At time 0, each sample exhibited distinct initial liposome diameters. *C. citratus* started with larger initial diameters, approximately 400 nm, while *C. nardus*, *C. commutatus* and *C. winterianus* had smaller initial sizes, measuring around 200 nm.

Over time, at 24 h, a continuous increase in liposome diameter was observed for *C. citratus* and *C. commutatus*, indicating particle growth or aggregation. In contrast, *C. winterianus* remained relatively stable throughout the observation period, showing minimal growth in diameter. This suggests that *C. winterianus* may be more stable than the other samples.

*C. citratus*, *C. commutatus* and *C. nardus* showed increasing diameters over 48 h. By 80 h, all three samples, *C. citratus*, *C. commutatus* and *C. nardus*, converged to a similar size, approximately 1000 nm, suggesting comparable endpoints in liposome growth.

This analysis highlights the differences in liposome stability and growth dynamics among the essential oil samples. *C. winterianus* demonstrated the highest stability, maintaining its size with minimal aggregation over time, in contrast to the progressive growth observed in the other formulations.

### 2.3. Obtention and Quantification of EO, Water-Soluble Part and Non-Soluble Water Part

The aerial parts of the fresh plant *C. commutatus* were extracted by hydrodistillation and the yield of essential oil was 1 g by 100 g of plant dried weight.

For the other EO, we used commercially available essential oils.

To explain the biological results, we analyzed the chemical composition of the total essential oil (EO), the water-soluble fraction, and the non-water-soluble fraction using gas chromatography–mass spectrometry (GC-MS).

For this purpose, a two-step extraction procedure was conducted. First, the essential oil was vigorously vortexed with water, resulting in the formation of two distinct phases. The upper phase, containing the non-water-soluble EO, was carefully removed and analyzed. The remaining water-soluble fraction was then extracted twice using the volatile solvent pentane.

The collected fractions were dried and evaporated under reduced pressure. The resulting residues were dissolved in hexane and subsequently analyzed by GC-MS.

**Figure 3:**
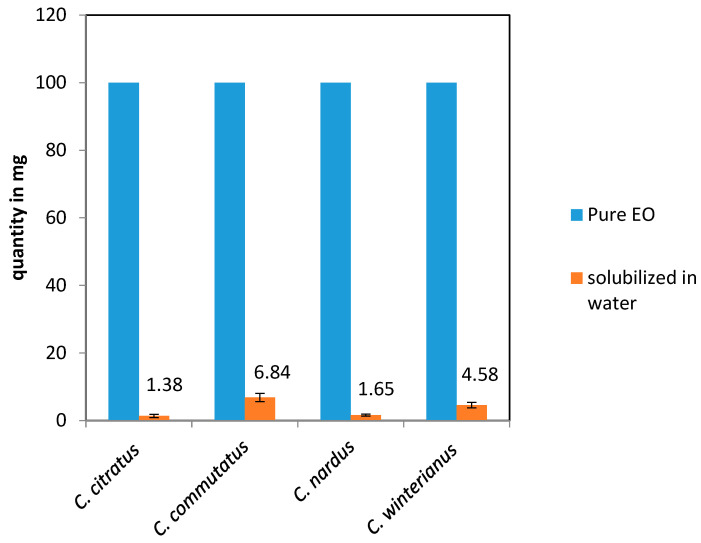
Water-solubilized fraction (in mg) of essential oils from four *Cymbopogon* species (*C. citratus*, *C. commutatus*, *C. nardus*, and *C. winterianus*), following water extraction.

Quantitatively, the water-soluble fraction extracted was significantly smaller compared to the total amount of pure EO used. Specifically, out of 100 mg of pure essential oil, 1.38 ± 0.2 mg, 6.84 ± 1.1 mg; 1.65 ± 0.3 mg, and 4.58 ± 0.3 mg residues of the water-soluble part were obtained for *C. citratus*, *C. commutatus*, *C. nardus*, and *C. winterianus, respectively (*Figure 3).

### 2.4. Phytochemical Composition of the EO, Water-Soluble Fraction and Non-Soluble Water Fraction by GC-MS

The chemical composition of the essential oils, as well as their water-soluble and non-water-soluble fractions, was analyzed by GC-MS. Compounds were considered when their relative abundance was ≥0.1% and their spectral similarity ≥ 92% (Figures S6–S17). All results are summarized in Table 2 and Table S1.

GC-MS analysis revealed 34 compounds in *C. citratus* essential oil, 17 in *C. commutatus*, 39 in *C. nardus*, and 31 in *C. winterianus*. Five compounds were common to all four essential oils. *C. citratus* displayed the highest number of unique compounds (21 not found in any other oil), while *C. commutatus* contained only 6 unique constituents.

In the water-soluble fraction of *C. citratus*, 20 compounds were identified, compared to 26 in the non-water-soluble fraction (Table 2). The water-soluble fraction of *C. commutatus* contained 16 compounds, while its non-water-soluble fraction showed the same compounds plus one additional constituent—piperitone—which was not detected in the water-soluble portion. In *C. nardus*, 35 compounds were found in the water-soluble fraction and 39 in the non-water-soluble fraction. Both the water-soluble and non-water-soluble fractions of *C. winterianus* contained 28 compounds each. The fractions did not share the following metabolites: citronellyl 2-butenoate, (3Z,5Z)-3,5-octadiene, geraniol formate, nerol acetate, elemene, cadinene, α-pinene epoxide and 10-epi-elemol, totaling eight compounds.

Qualitatively, the analysis of the water-soluble fraction of the four EOs shows the presence of almost all the pure EO constituents.

According to Table 2, the compounds identified in the pure essential oils are also present in both the water-soluble and non-water-soluble fractions for all four *Cymbopogon* species, although their relative abundances differ.

In the non-water-soluble fraction of *C. citratus*, trans-β-ocimene, myrtanal, limonene oxide, α-bergamotene, and β-elemol were identified. Isoeugenol was also detected exclusively in the non-water-soluble fraction of *C. winterianus*.

For the EO of *C. commutatus*, all the metabolites identified are present also in the water-soluble fraction and in the non-water-soluble fraction except for the piperitone detected only in this last phase.

However, some compounds were found exclusively in the non-water-soluble fraction after water extraction. This is the case for tricyclene, pinene, camphene, and β-myrcene, which were detected only in the non-water-soluble fraction of *C. nardus*. Palmitaldehyde and β-cubebene are the two non-shared metabolites identified in EO of *C. winterianus*.

*C. commutatus* exhibited the highest relative abundance of β-elemol, with 19% in the water-soluble fraction and only 5% in the non-water-soluble fraction (Table 2). In *C. winterianus*, β-elemol was also significant, representing 7.9% in the total EO, 7.4% in the water-soluble fraction and 8% in the non-water-soluble fraction. *C. nardus* and *C. citratus* showed much lower levels of β-elemol, with a maximum abundance of 1.5% and 0.3%, respectively, in the total EO.

*C. winterianus* displayed the highest levels of elemene, with 4.2% in the total EO and 3.6–4.3% in the water-soluble and non-water-soluble fractions. *C. commutatus* contained moderate amounts of elemene, with 2% in the water-soluble fraction and 0.5% in the non-water-soluble fraction. Lower concentrations were observed in *C. nardus* and *C. citratus*, where elemene levels were below 1.1%.

The highest concentration of geraniol acetate was observed in *C. nardus*, reaching 6.5% in the total EO and 3.3–6.1% in the water-soluble and non-water-soluble fractions. *C. winterianus* also contained notable amounts of geraniol acetate, with 4.8% in the total EO and 4.5–5% in the fractions. *C. citratus* and *C. commutatus* showed lower levels, with maximum values of 3.9% and 1.8%, respectively.

*C. nardus* EO contained the highest concentration of geraniol, with 28% in the water-soluble fraction, indicating it as a dominant compound in the EO of this sample. *C. citratus* EO showed a moderate amount of geraniol (5.8%), while *C. commutatus* EO had a minimal concentration of 0.3%.

Piperitone was predominantly found in *C. commutatus* EO, with an exceptionally high concentration of 73.9%, making it the defining compound for this sample. Notably, piperitone was absent in *C. nardus* EO and *C. winterianus* EO. A trace of this compound was found in *C. citratus* EO (0.1%).

Limonene was present in all samples but varied significantly in concentration. *C. nardus* EO contained the highest amount (8.6%), followed by *C. citratus* EO (6.7%). In contrast, *C. commutatus* EO and *C. winterianus* EO contained lower concentrations of limonene (2.7% and 2.1%, respectively).

The five compounds shared among the four essential oils are as follows: (±)-(R)-limonene, β-elemol, elemene, geraniol and geraniol acetate.

**Figure 4:**
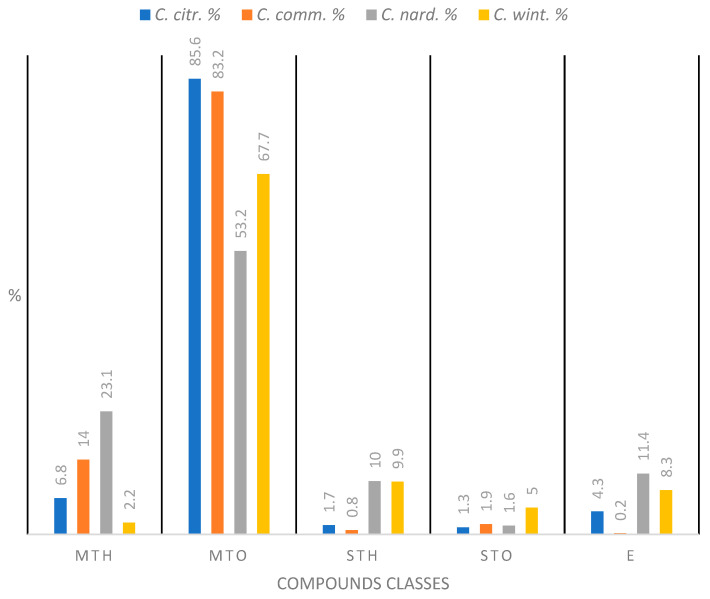
Relative abundance (%) of different classes of compounds identified in four essential oils (EOs): *Cymbopogon citratus* (*C. citr.*), *Cymbopogon commutatus* (*C. comm.*), *Cymbopogon nardus* (*C. nard.*), and *Cymbopogon winterianus* (*C. wint.*). MTH: hydrocarbonated monoterpenes, MTO: oxygenated monoterpenes, STH: hydrocarbonated sesquiterpenes, STO: oxygenated sesquiterpenes and E: esters.

The chemical composition of the four essential oils (EOs), from *Cymbopogon citratus* (*C. citr.*), *Cymbopogon commutatus* (*C. comm.*), *Cymbopogon nardus* (*C. nard.*), and *Cymbopogon winterianus* (*C. wint.*), reveals distinct differences in the abundance of compound classes (Figure 4).

Oxygenated monoterpenes (MTOs) are the most abundant class in the essential oils studied in comparison to the other classes of compounds (Table 2). This family represents 85.6%, 83.2%, 53.2%, and 67.7% of the constituents of *C. citratus*, *C. commutatus*, *C. nardus*, and *C. winterianus,* respectively (Figure 4).

*C. nardus* contains hydrocarbonated monoterpenes (MTHs), representing nearly 23.1% of its composition. In contrast, the other species (*C. commutatus*, *C. citratus*, and *C. winterianus*) show significantly lower levels, all below or equal to 14%. The class of hydrocarbonated sesquiterpenes (STHs) is present in relatively small amounts across all species, not exceeding 10% in any of the tested oils.

*C. winterianus* exhibits the highest proportion of oxygenated sesquiterpenes (STOs) (5%). The other EOs showed STO levels between 1.3 and 1.9%. Esters (Es) are detected in all EOs.

### 2.5. Determination of Antibacterial Activity of Pure Compounds

Table 3 presents the minimum inhibitory concentrations (MICs) of four pure compounds against seven bacterial strains, including both Gram-positive and Gram-negative bacteria.

The compounds tested include two shared compounds in the different EO, in particular geraniol and limonene ((+)-(R)-limonene and (−)-(R)-limonene). Piperitone, the major compound found in *C*. *commutatus* EO, was also tested. The MIC values indicate the concentration at which the compounds inhibit bacterial growth.

Piperitone exhibited very strong antibacterial activity, with an MIC of <0.04% while geraniol demonstrated moderate antibacterial activity against *S. aureus* with an MIC of 0.63%. In contrast, the limonene isomers, (+)-(R)-limonene and (−)-(R)-limonene, did not exhibit any antibacterial effect, with MICs greater than 5%. A translucent surface film appeared at high concentrations, particularly for (+)-(R)-limonene and (−)-(R)-limonene experiments. Beneath this layer, the medium remained liquid and could be pipetted.

Geraniol exhibited moderate antibacterial activity with an MIC of 0.31% against *P. aeruginosa* and *A. baumannii*, while both limonene isomers showed no activity, with MICs greater than 5%. Piperitone demonstrated an MIC of 1.25% against *P. aeruginosa*, suggesting lower effectiveness against this strain comparatively against *S. aureus*.

Against the strain *E. coli* and *E. cloacae*, only geraniol showed an inhibitory activity with an MIC of 0.63% and 2.5%, respectively.

Geraniol displayed moderate activity against several bacterial strains, including *S. aureus*, *E. faecalis*, *P. aeruginosa*, *A. baumannii* and *E. cloacae* with MICs ranging from 0.31% to 2.5%.

### 2.6. Cytotoxicity Activity

In this study, *Cymbopogon* essential oils (*C. citratus*, *C. commutatus*, *C. nardus*, and *C. winterianus*) exhibited strong cytotoxic effects at both MICs and 2× MICs, reducing cell viability to nearly zero. In contrast, liposomal lecithin maintained significantly higher viability (80% at MIC and 60% at 2× MIC), suggesting a markedly lower cytotoxic profile.

## 3. Discussion

Numerous essential oils exhibit antimicrobial activity against Gram-negative bacteria such as *Acinetobacter baumannii*, *Escherichia coli*, *Klebsiella pneumoniae*, and *Pseudomonas aeruginosa*, which are responsible for infections that are increasingly difficult to treat due to the emergence of multidrug resistance as reported by Aelenei [19].

Recent research has highlighted the diverse bioactivities of *Cymbopogon* essential oils, showcasing their potential applications in pharmaceuticals, food preservation, and aromatherapy [15,16,17]. Most pharmacological studies have been conducted in the Indian subcontinent, with a predominant focus on the essential oils of plants from this region. These studies have demonstrated various biological effects, including an anti-inflammatory effect for *Cymbopogon giganteus* essential oil [20], an anticancer effect for *Cymbopogon schoenanthus* essential oil [21], an antiviral effect for *C. nardus* essential oil [22], and an antipyretic effect for *C. citratus* essential oil [23].

**Encapsulation** is a technique used to protect and to solubilize essential oils. Several reviews have summarized the various techniques for the encapsulation of essential oils, highlighting advancements in the field [7,8,9,10]. Among essential oils, *Cymbopogon citratus* (lemongrass) is the most widely studied for encapsulation due to its significant biological activities.

Various encapsulation methods have been applied to *Cymbopogon citratus* essential oil, such as liposomes [24], biopolymer matrices with ZnO nanoparticles [25], maltodextrin–gelatin microparticles [26], and spray-drying with gum Arabic [27], to enhance its stability and bioactivity. For *C. winterianus*, nanoemulsions formulated via cavitation [28] or with Tween 20 and butanol [29] showed antimicrobial effects, though comparisons are limited due to differing assay methods.

In our study, liposomes formulated with all tested essential oils (*C. citratus*, *C. commutatus*, *C. nardus*, and *C. winterianus*) exhibited strong antimicrobial activity.

In all cases, a significant decrease in MIC values was observed for the emulsions of the four EOs, indicating enhanced antibacterial activity compared to the water-soluble fraction alone (Table 1). The most notable effects were observed against Pseudomonas aeruginosa, where L + C. citratus and L + C. winterianus achieved MIC values of 0.08%. Both formulations were similarly effective against *Escherichia coli*, also with MICs of 0.08%. Against *Acinetobacter baumannii*, L + *C. nardus* showed the greatest inhibition (MIC: 0.04%). In addition, L + *C. citratus* inhibited *Enterobacter cloacae* with an MIC of 0.08%. Among Gram-positive bacteria, L + *C. winterianus citratus* demonstrated the strongest activity against *Staphylococcus aureus* (MIC: 0.02%), while both L + *C. citratus* (MIC: 0.08%) and L + *C. winterianus* (MIC: 0.04%) exhibited significant efficacy against *Enterococcus faecalis*.

This improved efficiency is not attributed to the liposome itself (inactive up to 25%) but rather to the fact that all EO compounds, including both hydrophilic and hydrophobic components, are effectively solubilized in the culture medium.

For example, the *C. citratus* microemulsion exhibited antibacterial activity against six bacterial strains, with *K. pneumoniae* being the only strain unaffected. MIC values ranged between 0.04% for *S. aureus* and 0.08% for *E. faecalis*, *P. aeruginosa*, *E. coli*, *A. baumannii*, and *E. cloacae*.

The size and stability measurements of the liposomes were performed within a maximum of 80 h after preparation. During this period, the liposome containing the essential oil of *C. winterianus* remained stable. (Figure 2). However, no phase separation was observed for all liposomes at 72 h. The incorporation of *C. citratus* essential oil led to larger liposomes (~400 nm), likely due to greater encapsulation of hydrophobic compounds in the lipid bilayer.

*C. winterianus* exhibits the greatest stability, with almost no increase in liposome diameter over 80 h, indicating better resistance to aggregation or environmental influences. In contrast, *C. citratus*, *C. commutatus*, and *C. nardus* appear less stable, as evidenced by significant particle growth over time, which suggests a tendency toward aggregation.

These observations have important implications for the formulation, storage, and application of these essential oils. The stability of *C. winterianus* suggests that it may possess a more suitable formulation or greater environmental compatibility, making it robust for long-term storage or applications requiring consistent stability. On the other hand, the increasing particle size observed in *C. citratus*, *C. commutatus*, and *C. nardus* may indicate limited stability in formulations. This could impact applications where consistent particle size is critical, such as drug delivery systems or controlled-release technologies.

To our knowledge, no studies have reported the formulation of liposomes using soybean lecithin alone for the encapsulation of *Cymbopogon* essential oils. Existing formulations typically combine soybean lecithin with other stabilizing agents such as cholesterol or surfactants to enhance liposome stability and performance. It is the case for essential oil from *C. densiflorus* leaves [30], ginger oil [31] or lavandin essential oil [32]. Comparison with our system is not possible due to differences in liposome formulation.

Encapsulation of *Cymbopogon commutatus* essential oil into chitosan nanoparticles has previously resulted in MIC values of 6% against *E. coli* and 1.58% against *S. aureus* [33]. In comparison, our formulation exhibited significantly lower MICs of 0.08% and 0.31%, respectively.

The analysis by GC-MS highlights the diversity in chemical composition among *Cymbopogon* species (Figure 4). All essential oils are dominated by oxygenated monoterpenes. EOs of *C. commutatus* and *C. nardus* exhibit a significant proportion of hydrocarbonated monoterpenes (14 and 23%, respectively). *C. nardus* shows a lower amount of oxygenated monoterpenes but the presence of all chemical classes. *C. commutatus* uniquely contains a very low amount of esters. These compositional differences may influence the biological activities of these essential oils, including their antimicrobial and therapeutic properties [34].

*Cymbopogon citratus* essential oil (EO), commonly known as lemongrass oil, is predominantly composed of citral (70–85%), followed by myrcene (10–20%), limonene (1–5%), geraniol (2–5%), and trace amounts of citronellal [23,35,36]. Citral is the key compound responsible for the characteristic lemon scent of *C. citratus* EO and is highly valued for its antimicrobial and anti-inflammatory properties [37]. The EO of *C. citratus* has been shown to be highly effective against a variety of bacterial and fungal pathogens. Studies highlight the strong antibacterial activity of citral, particularly against *Staphylococcus aureus* and *Escherichia coli* [38]. Additionally, the EO demonstrates significant antifungal effects, particularly against *Candida albicans* [39].

The primary compounds in *Cymbopogon nardus* EO (Ceylon Citronella) include citronellal (30–35%), geraniol (18–25%), citronellol (10–15%), limonene (5–7%), and eugenol (1–3%) [40]. *C. nardus* EO is widely known for its use as a natural insect repellent. Beyond its repellent properties, it exhibits antimicrobial activity, being effective against both Gram-positive and Gram-negative bacteria. This variation across samples highlights *C. nardus* as the most significant source of geraniol, a compound known for its antioxidant and antimicrobial properties [40,41].

The essential oil of *Cymbopogon winterianus* (Java Citronella) is composed of citronellal (30–45%), geraniol (20–30%), citronellol (10–20%), limonene (5–10%), and traces of nerol [42]. *C. winterianus* produces citronella oil, which is widely recognized for its insect repellent properties, particularly against mosquitoes [43]. In addition to its repellent effects, *C. winterianus* EO demonstrates antifungal and antibacterial properties. It is also effective as an insecticide against various pests [44]. The essential oil has shown significant activity against fungal pathogens such as *Candida albicans* and *Aspergillus niger* [45,46].

*Cymbopogon commutatus*, commonly known as “Camel Grass” or “Dhofar Grass”, has been the subject of limited studies focusing on its chemical composition and biological activities. This research is far less extensive compared to other Cymbopogon species, such as lemongrass (*Cymbopogon citratus*) and citronella (*Cymbopogon winterianus*).

*C. commutatus* (*Steud.*) Stapf, used in our study, is a wild plant native to Djibouti, commonly known as “caws dameer”, where it is traditionally used in folk medicine. In decoctions, it is administered alone or with tea to treat conditions such as kidney problems, jaundice, and bladder inflammation [47]. Similar preparations are used in Pakistan for the treatment of headaches and fever [48], while in Egypt and Saudi Arabia, the essential oil of this species is valued as a natural insecticide [49,50]. Comparable to other *Cymbopogon* species, *C. commutatus* essential oil (EO) has been shown to be an effective natural insect repellent, particularly against mosquitoes, due to its significant citronellal content [17].

The current study marks the first time the essential oil of *C. commutatus* from Djibouti has been chemically and biologically analyzed.

Notably, *C. commutatus* EO exhibited the highest amount of water-soluble compounds 6.84 ± 1.1 mg/100 mg compared to the other essential oils.

The essential oil of *C. commutatus* typically contains a variety of monoterpenes and sesquiterpenes, contributing to its aromatic and biological properties.

In the literature, the most common components identified include limonene, citronellal, geraniol, citral, and terpinen-4-ol [33,51]. Limonene is known for its citrus scent and potential antimicrobial properties [52]. Citronellal contributes to the oil’s lemony aroma and is widely used as a natural insect repellent [43]. Geraniol is characterized by its sweet, rose-like scent. It is commonly used in perfumes and flavorings and possesses antimicrobial and antioxidant properties [53]. Citral is a compound with a strong lemon scent, widely utilized in flavoring, fragrances, and as an antimicrobial agent [54]. Terpinen-4-ol is a terpene known for its anti-inflammatory and antimicrobial activities [3].

The biological activity of *C. commutatus* essential oil has been primarily investigated for its antimicrobial, antioxidant, and insecticidal properties. The oil has demonstrated efficacy against a variety of bacterial and fungal pathogens, with its antimicrobial action largely attributed to its high citral and geraniol content, which are known for their ability to disrupt microbial cell membranes [54,55].

Notably, our analysis revealed that *C. commutatus* EO is exceptionally rich in piperitone, comprising 73% of the total composition. This unique abundance suggests that piperitone may serve as a marker compound for *C. commutatus*, distinguishing its chemical profile from other *Cymbopogon* species.

Piperitone is a monoterpene ketone found in other essential oils, including those from Eucalyptus [4] and certain Mentha species [56]. Piperitone has demonstrated several biological activities such as antimicrobial properties, antioxidant properties and insecticidal effects [57]. Piperitone is particularly effective against bacterial strains. For instance, studies have shown that piperitone reduces the resistance of certain enterobacteria to nitrofurantoin, enhancing the antibiotic’s effectiveness [58]. It contributes to the neutralization of free radicals, thereby protecting cells from oxidative stress. Research indicates that piperitone exhibits significant activity against harmful insects, making it a promising candidate for natural pest control [57].

These findings provide a strong foundation for the potential therapeutic applications of *Cymbopogon commutatus* essential oil, both in traditional medicine and modern applications such as antimicrobial treatments, insect repellents, and antioxidants.

In our study, piperitone (73.9%), a water-insoluble compound and the major constituent of *C. commutatus* essential oil, showed very strong antibacterial activity against *Staphylococcus aureus* and strong antibacterial activity against *Pseudomonas aeruginosa* with an MIC of <0.04% and 1.25%, respectively.

The weak activity of the water-soluble fraction of *C. commutatus* against *S. aureus* (MIC: 25%) may be explained by the absence of piperitone. In contrast, the liposomal formulation exhibited enhanced activity (MIC: 0.08%), consistent with the high efficacy of pure piperitone (MIC < 0.04%).

In the case of analysis against *S. aureus*, a vapor-phase effect was observed on the growth controls located near wells with high product concentrations, with no visible bacterial growth. In contrast, controls located near wells containing lower concentrations exhibited normal visible growth. The other rows of the plate, seeded with medium and bacteria only (without piperitone), also exhibited this effect: wells located near those containing high concentrations of the product showed no bacterial growth.

This effect may suggest a potential vapor-phase diffusion of volatile compounds, possibly leading to growth inhibition in neighboring wells. The observed absence of growth in untreated control wells located near high-concentration samples could be due to indirect exposure. However, further investigation would be necessary to confirm this hypothesis. These findings underline the importance of considering vapor-mediated interactions when designing microplate-based assays involving volatile substances.

For the other bacterial strains, the results could not be determined due to the opacity of the solution.

At high concentrations, a translucent surface film was observed, especially with the (+)-(R)-limonene and (−)-(R)-limonene experiments. The underlying medium remained liquid and could still be pipetted. This phenomenon likely affected the results.

Our data suggest that while some compounds like geraniol and piperitone show promise as antibacterial agents, the limonene isomers require further exploration or may not be as effective.

**Figure 5:**
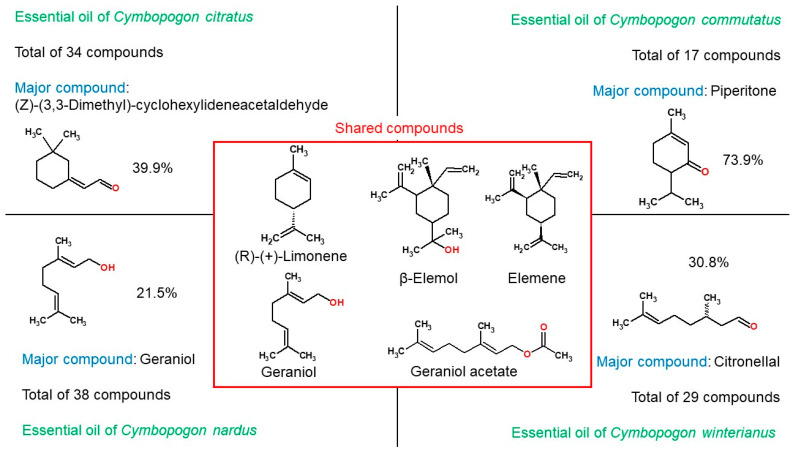
Major compounds identified using GC-MS and shared molecules in the four essential oils studied: (±)-(R)-limonene, α-elemol, elemene, geraniol and geraniol acetate.

In Figure 5, we highlight the unique and variable presence of (±)-(R)-limonene, α-elemol, elemene, geraniol, and geraniol acetate across the studied samples.

Several studies have demonstrated that *Cymbopogon* essential oils, particularly those rich in citral and geraniol, exhibit strong antimicrobial activity against bacterial and fungal pathogens [42]. Citral showed a lower MIC (0.14%) against *A. baumannii* compared to *Cymbopogon flexuosus* oil (0.65%) using the microtiter plate method [59]. The presence of compounds such as limonene, citronellal, and geraniol also contributes to the oils’ effectiveness against pathogens like *Staphylococcus aureus* and *Candida albicans* [60,61].

A major challenge in this evaluation is the poor solubility of EOs in bacterial culture media, which are predominantly water-based. This issue has been widely acknowledged in the literature, and several studies have attempted to address it using EO dispersants.

Many essential oils (EOs) are hydrophobic (water-insoluble), which limits their direct application in aqueous environments such as biological systems. Liposomes, with their phospholipid bilayer structure, can encapsulate both hydrophilic and hydrophobic compounds, enabling essential oils to disperse more effectively in biological fluids and improving their bioavailability [11,62].

The essential oil (EO)/liposome system demonstrates improved antimicrobial activity due to the complete solubilization of all EO compounds, both hydrophilic and hydrophobic, in the aqueous phase, resulting in better dispersion.

The observed differences in liposome size may be influenced by the chemical composition of the essential oils, particularly their hydrophobicity. Liposomes formulated with *C. citratus* essential oil were significantly larger than those prepared with *C. commutatus*, *C. nardus*, and *C. winterianus*. This may be related to the higher proportion of hydrophobic compounds in *C. citratus*, as suggested by its higher average logP value, which could favor greater incorporation into the lipid bilayer and contribute to increased particle size. For essential oils (EOs), antimicrobial susceptibility testing (AST) is limited by the poor solubility of EOs in the liquid medium used for determining antibacterial activity. As a result, these tests are conducted only on the water-soluble components of essential oils, meaning the observed activity may not fully represent all compounds present in the EOs.

The chemical nature of the major constituents in pure essential oils (EOs) plays a critical role in determining their degree of solubilization in water. A key parameter that reflects a compound’s affinity for water is the partition coefficient (P), which describes its distribution between an aqueous phase and an immiscible organic solvent. The logarithmic value of this coefficient (log P) indicates the compound’s polarity: a positive log P denotes hydrophobicity, whereas a negative value indicates hydrophilicity. Log P values for most compounds are readily available in the literature [63]. The specific compounds identified in our four essential oils are summarized in Table 4.

The Log P values of these compounds are positive, indicating their hydrophobic nature. The hydrophobicity of the major compounds identified in the *Cymbopogon* essential oils, as reflected by their log P values, likely plays a key role in their distribution between water-soluble fractions and liposomal formulations. Compounds such as (Z)-(3,3-dimethyl)-cyclohexylideneacetaldehyde (log P = 2.71) and piperitone (log P = 2.85) exhibit moderate hydrophobicity and may therefore be partially present in both aqueous and lipidic environments. In contrast, compounds such as geraniol, citronellal, and β-citral, with log P values ranging from 3.2 to 3.45, are more hydrophobic and tend to localize preferentially within lipid bilayers. Highly hydrophobic molecules such as camphene (log P = 4.22), (+)-2-carene (log P = 4.32), and (±)-(R)-limonene (log P = 4.57) are expected to be almost exclusively associated with the liposomal phase. These variations in compound partitioning may influence the antimicrobial efficacy of the formulations.

As discussed above, the antibacterial tests were conducted using the water-soluble fraction of the essential oils (EOs). Therefore, the observed MIC values may not fully represent the activity of all compounds present in the EOs, particularly from a quantitative perspective.

Essential oils (EOs) can exhibit potent antibacterial properties, but they may also be toxic when misused or administered in high concentrations [54]. Studies on the toxicity of *Cymbopogon* EOs remain limited, with only *C. citratus* currently classified as Generally Recognized As Safe (GRAS) by the U.S. FDA [64]. In our study, strong cytotoxic effects were observed for *Cymbopogon* EOs (*C. citratus*, *C. commutatus*, *C. nardus*, *C. winterianus*) at their minimum inhibitory concentrations (MICs) and at twice the MICs, with cell viability reduced to nearly zero. In contrast, liposomal lecithin showed significantly lower cytotoxicity, with cell viability maintained at 80% at MIC and 60% at 2× MIC, suggesting a protective effect of the liposome formulation [13].

## 4. Materials and Methods

### 4.1. Chemicals

(R)-(+)-Limonene, (S)-(+)-limonene, piperitone, geraniol, and DMSO were purchased from Sigma Aldrich (Saint Quentin Fallavier, France). 1,2 Diacyl-sn-glycero-3-phosphorylcholine (soybean lecithin) is obtained from ACROS Organics, part of Thermo Fischer (Illkirch, France).

### 4.2. Starting Materials and Preparation of the Essential Oil (EO)

The plant *Cymbopogon commutatus* was collected in 2015 from Lac Asal, located in the northern region of Djibouti. The sample was authenticated by Professor Maha Kordofani from the University of Khartoum, Sudan. The voucher specimen No. 48/HND/2015 was deposited at the National Herbarium of Djibouti.

The aerial parts of the fresh plant (700 g) were used to extract essential oil through hydrodistillation. The extracted oil was dried using Na_2_SO_4_, filtered, and stored at −18 °C prior to chemical and biological analyses.

The other three essential oils of *Cymbopogon* species were commercially sourced and purchased from a pharmacy: EO from *Cymbopogon citratus* (origin Guatemala, Phytosun aroms, Laboratoire Perrigo, Châtillon, France), EO from *Cymbopogon nardus* (origin Sri Lanka, Phytosun aroms, Laboratoire Perrigo, Châtillon, France) and EO from *Cymbopogon winterianus* (origin Indonesia, Naturactive, Laboratoire Pierre Fabre, Castres, France).

The acronyms used in the text are as follows: *C. citratus* is *C. citr.*, *C. commutatus* is *C. comm.*, *C. nardus* is *C. nard*. and *C. winterianus* is *C. wint*.

### 4.3. Evaluation of Antibacterial Activity

Seven bacterial strains were tested, including two Gram-positive strains (*Staphylococcus aureus* ATCC 29213 and *Enterococcus faecalis* CIP 103214) and five Gram-negative strains (*Pseudomonas aeruginosa* ATCC 27853, *Escherichia coli* ATCC 25922, *Klebsiella pneumoniae* CIP 110855, *Acinetobacter baumannii* non-resistant clinical strain, and *Enterobacter cloacae* non-resistant clinical strain).

All experiments were conducted using freshly prepared bacterial suspensions incubated for 18 h at 35 °C in Mueller–Hinton Broth, Cation-Adjusted (MHB-CA). The minimum inhibitory concentration (MIC) values of the samples were determined using the microdilution method in accordance with the ISO 20776-1 standard [18], with specific adaptations.

For evaluation of pure essential oil, deionized water was added to pure essential oil (EO) in a volume/volume ratio, followed by gentle mixing through three repeated inversions. After a decanting period of 2 h, the water-soluble fraction was collected and serially diluted at a ratio of 1:2 in MHB-CA. The bacterial inoculum was then added to reach a final volume of 100 µL and a bacterial concentration of 5 × 10^5^ CFU/mL, with a sample dilution range from 25% to 0.1% (*v*/*v*). Controls included sterility control (MHB-CA + 25% water), product control (MHB-CA + 25% sample), and growth control (bacteria in MHB-CA + 25% water).

The controls performed on each plate were: medium-only control, medium + sample control (25% *v*/*v*), and bacteria control (growth control).

A range of 1/2 serial dilutions was tested for each sample (from 25% to 0.1% *v*/*v*).

The bacterial inoculum was prepared to achieve a final concentration of 5 × 10^5^ [2 × 10^5^–8 × 10^5^] CFU/mL in the wells.

Growth detection was performed visually at 24 h.

For pure compounds, each test was performed once using the microdilution method in Mueller–Hinton 2 broth, with a final volume of 100 μL per well. The controls included on each plate were as follows: a sterility control, a medium + pure compound control (5% *v*/*v*), and a bacterial growth control. A two-fold serial dilution series was tested for each compound, ranging from 5% to 0.4% (*v*/*v*). No dispersing agent was used. For serial dilutions, each dilution (X) was mixed by aspirating and dispensing ten times before transferring to the next dilution (X + 1). Bacterial growth was assessed after 24 h of incubation at 35 °C by visual observation of growth presence or absence in each well.

For (±)-(R)-limonene, geraniol, and piperitone, the tested range was adjusted to 5% to 0.04% (*v*/*v*), and tests were performed in triplicate (*n* = 3).

The encapsulated essential oils appeared white and opaque, with a milky aspect. Each MIC was determined using the microdilution method in Mueller–Hinton 2 broth, with a final volume of 100 μL per well. The controls included on each plate were as follows: medium-only control, medium + sample control, and bacterial control (growth control). A two-fold serial dilution range was tested for each sample.

The bacterial inoculum was prepared to achieve a final concentration of 5 × 10^5^ CFU/mL [range: 2 × 10^5^ to 8 × 10^5^ CFU/mL] in wells 1, 2, and 3. Bacterial growth was assessed after 24 h by visually observing the presence or absence of growth in the wells. The tested range was 25% to 0.1% (*v*/*v*), corresponding to 1.25% to 0.005% of encapsulate EO, with each test performed eight times (*n* = 8). All analyses were carried out within 48 h following the preparation of the liposomes.

### 4.4. Preparation and Characterization of Liposome

The four essential oils (EOs) were encapsulated into liposomes as described in the following method [65]. A total of 30 mg of soy lecithin was dissolved in chloroform, and the solvent was evaporated to form a thin layer of lecithin at the bottom of the flask. After complete drying, 0.5 mL of *Cymbopogon* essential oil and 10 mL of water were added to the flask. The resulting mixture was sonicated for 30 min using a Bandelin Sonoplus HD2200.

The stability and homogeneity of the formulated liposomes were monitored using a Zetasizer 3000HSA (Malvern Pananalytics, Malvern, UK), which recorded the distribution of liposome dimensions for the samples (*C. citratus*, *C. commutatus*, *C. nardus*, and *C. winterianus*). The Contin algorithm, which accounts for the polydispersity of the sample, was applied for the analysis.

The key parameters measured using dynamic light scattering (DLS) included the following: particle size distribution and polydispersity index (PDI), run parameters such as angle, KCps, ZAve, polydispersity index (Poly), and fit times, average measurements for size, intensity, volume, and number across multiple runs and peak analysis based on intensity, volume, and number [66].

### 4.5. Preparation of the Samples for GC-MS Analysis and Equipment

To investigate the chemical composition of essential oils (EO), two types of samples were prepared:

(A) The essential oils and pure compounds were directly diluted with hexane (50 μL/mL) and analyzed by GC-MS.
(B) An equal mixture of EO and water was vortexed to separate the non-water-soluble and water-soluble fractions. The upper phase, consisting of the non-water-soluble EO, was carefully removed. The remaining water-soluble fraction was extracted twice with pentane. The collected organic layers were dried and evaporated under reduced pressure. The resulting residues were dissolved in hexane, and both the non-water-soluble and water-soluble parts were analyzed by GC-MS.

For each solution (EO, water-soluble phase and non-water-soluble phase), 1 μL was injected into a Shimadzu QP 2010 GC-MS system (Shimadzu, Kyoto, Japan) equipped with a DB-5 ms column (30 m, 0.25 mm thickness). The temperature program for the analysis was as follows: an initial hold at 60 °C for 5 min, followed by a temperature ramp up to 320 °C over 36 min. Helium was used as the mobile phase at a flow rate of 1.0 mL/min, and the injector temperature was set at 250 °C.

The mass spectrometer operated in electron impact (EI) mode, scanning in the *m*/*z* range of 35 to 900. Compound identification was achieved by comparing the individual mass spectra of each compound to those stored in the Shimadzu NIST08 library. Further confirmation of the identified compounds was carried out by comparison with reference spectra available in the NIST database. To ensure consistency and reliability in compound annotation, we applied the following criteria: (i) relative abundance ≥ 0.1% of total chromatographic area, (ii) consistent retention time across replicates, and (iii) spectral similarity ≥ 92%, with attention to the characteristic fragmentation pattern of each molecule.

### 4.6. Determination of Cytotoxicity

The toxicity of essential oils (EOs) on healthy lung cells MRC-5 (ATCC, CCL-171) was assessed using the MTT test [67], as derived from Mosmann [68]. MRC-5 is a human embryonic fibroblast cell line commonly used as a model for toxicity evaluation. The assay is based on the reduction of MTT into formazan crystals by the cellular metabolism of viable cells.

MRC-5 cells were plated at 10,000 cells per well in a complete medium (Minimum Essential Medium (MEM), Sigma-Aldrich, M4655 + 10% fetal calf serum) in 96-well tissue culture plates and incubated for 48 h at 37 °C. Following this, the medium was discarded and replaced with fresh medium containing the test sample (pure essential oils or liposomes). Each condition was tested in four technical replicates. Three different controls were included, i.e., medium alone, cells in medium, and sample in medium.

After a 24 h incubation period, the medium was replaced with 100 µL of fresh medium containing 0.5 mg/mL MTT per well, and the plates were incubated for an additional 4 h at 37 °C. The resulting formazan crystals were dissolved using 100 µL of dimethyl sulfoxide (DMSO). Absorbance was measured at 540 nm using a 96-well plate reader. Cell survival percentages were calculated using MS Office Excel.

### 4.7. Statistical Analysis

The measurements were repeated three times, and the data are expressed as the mean ± standard error of the mean (SEM). The Contin algorithm was used to describe the size distribution of the EO emulsions.

## 5. Conclusions

We report, for the first time, the formulation of liposomes using soybean lecithin alone for the encapsulation of *Cymbopogon commutatus* essential oil from Djibouti, along with three commercially available essential oils derived from other *Cymbopogon* species (*C. citratus*, *C. nardus*, and *C. winterianus*).

- The antibacterial activity of encapsulated essential oils using natural soy lecithin-based liposomes was significantly enhanced. For instance, the MIC decreased from 25% (water-soluble EO fraction) to 0.02% for liposome-encapsulated *C. winterianus* EO against *S. aureus*, and to 0.04% for *E. faecalis*.
- GC-MS analysis revealed that piperitone was the major compound in *C. commutatus* EO, with a relative abundance of 73.9%. This compound exhibited strong antibacterial activity, with an MIC < 0.04% against *S. aureus*.
- All four *Cymbopogon* essential oils shared five common compounds: (±)-(R)-limonene, β-elemol, elemene, geraniol, and geraniol acetate.

## Figures and Tables

**Table 1:** Antibacterial activity of four water-soluble fraction and formulate essential oils with soybean lecithin liposome.

Bacteria Strains		*C. citr.* %	*C. comm.* %	*C. nard.* %	*C. wint.* %	Liposome alone (L) %	L + *C. citr.* %	L + *C. comm.* %	L + *C. nard.* %	L + *C. wint.* %
Gram-positive	*S. aureus*	25	25	>25	25	>25	0.04	0.08	0.08	0.02
	*E. faecalis*	>25	25	>25	25	>25	0.08	>0.16	>0.16	0.04
Gram-negative	*P. aeruginosa*	>25	>25	>25	>25	>25	0.08	0.31	0.16	0.08
	*E. coli*	25	>25	>25	>25	>25	0.08	0.31	>0.16	0.08
	*K. pneumoniae*	>25	>25	>25	>25	>25	>0.16	>0.16	>0.63	>1.25
	*A. baumannii*	25	25	>25	25	>25	0.08	0.16	0.04	>0.63
	*E. cloacae*	>25	25	25	>25	>25	0.08	>0.16	>0.16	>0.63

The MIC values are expressed as % (*v*/*v*) of the samples tested against the target strains. For *C. citratus*, *C. commutatus*, *C. nardus*, and *C. winterianus*, the MIC was determined using the water-soluble fraction. For L + *C. citratus*, L + *C. commutatus*, L + *C. nardus*, and L + *C. winterianus*, the MIC was determined using the pure essential oils formulated with liposomes. Results are based on eight replicates (*n* = 8).

**Table 2:** Chemical composition of pure essential oil (EO), of water-soluble and non-water-soluble fractions in the four essential oils of *Cymbopogon* species.

			*C. citratus* (*C. citr.*)			*C. commutatus* (*C. comm.*)			*C. nardus* (*C. nard.*)			*C. winterianus* (*C. wint.*)		
RT (min)	Compound Name	Chemical Class	EO (%) *	Water- Soluble Fraction (%) *	Non-Water-Soluble Fraction (%) *	EO (%) *	Water-Soluble Fraction (%) *	Non- Water-Soluble Fraction (%) *	EO (%) *	Water-Soluble Fraction (%) *	Non- Water-Soluble Fraction (%) *	EO (%) *	Water- Soluble Fraction (%) *	Non- Water-Soluble Fraction (%) *
4.13	Tricyclene	MTH							1		1.1			
4.42	Pinene	MTH							2.2		2.1			
4.9	Camphene	MTH							7.8		7.4			
6.04	Sulcatone	MTO	0.7	0.8	0.6									
6.12	β-myrcene	MTH							0.6		0.8			
6.33	(+)-2-Carene	MTH				11.1	0.9	14						
7.13	(±)-(R)-Limonene	MTH	6.7	0.7	7	2.7	5.6	3.6	8.6	1.7	8.4	2.1	1.3	2.2
7.34	Trans-β-Ocimene	MTH	0.1		0.1				1.5	0.3	1.7			
7.59	Ocimene	MTH							0.8	0.1	0.9			
8.19	4-Nonanone	MTO	1	0.6	1									
8.44	α-Terpinolene	MTH							0.6	0.4	0.6			
8.78	β-linalool	MTO	1.2	1.7	1.2				0.6	1.4	0.7	0.4	0.8	0.5
8.88	Myrtanal	MTO	0.1		0.1									
9.26	(E)-*p*-2-Menthen-1-ol	MTO				1.7	5.1	2.1						
9.54	α-Pineneoxide	MTO	0.2	0.2	0.2									
9.62	(Z)-p-2-Menthen-1-ol	MTO				0.9	3.3	1.3						
9.73	*p*-Menth-8-en-3-ol	MTO							0.2	2.1	0.6	1.5	3	1.4
9.74	Trans-4,5-epoxycarene	MTO	0.2	0.2	0.2									
9.82	Citronellal	MTO	1.1	0.2	0.3				3.7	2.5	3.8	30.8	25.6	30
9.92	(-)-Isopulegol	MTO	0.5	0.2	0.5							0.6	0.7	0.5
9.99	1,3,4-trimethyl-3-Cyclohexene-1-carboxaldehyde	MTO	0.5	0.2	0.5									
10.11	Camphenol	MTO							0.3	1	0.1			
10.17	(+)-Borneol	MTO							7.3	15.3	6.7			
10.33	Trans-4,5-epoxy-carane	MTO	1.1	1	1									
10.33	Terpinen-4-ol	MTO							1.1	3.2	1			
10.54	(3Z,5Z)-3,5-Octadiene	MTH	0.1											
10.58	Isopinocampheol	MTO	0.3	0.8	0.3									
10.59	α-Terpineol	MTO				2.1	14	2.8	1.6	5	1.5			
10.79	Decanal	MTO	0.2	0.2	0.2									
10.81	Palmitaldehyde	MTO										0.1		
10.83	Trans-Piperitol	MTO				0.6	2.4	0.9						
11.09	Geranyl nitrile	MTH	0.1		0.1									
11.11	Citronellol	MTO							4	3.8	4.2	11.4	12.1	11.6
11.32	Neral	MTO										0.3	0.5	0.3
11.37	β-Citral	MTO	32.5	38.6	33									
11.51	Geraniol	MTO	5.8	6.7	4.9	0.3	1	0.2	21.5	28	18.5	20.4	22.3	19.8
11.6	Piperitone	MTO	0.1	0.5	0.3	73.9		62						
11.8	α-Citral	MTO							0.4	1	0.5	0.6	0.9	0.6
11.85	(Z)-(3,3-Dimethyl)-cyclohexylideneacetaldehyde	MTO	39.9	43.4	41.3									
12.03	Borneol acetate	E							0.7	0.4	0.7			
12.03	Limonene oxide	MTO	0.2		0.2									
12.24	Geraniol formate	E	0.1						0.4	0.3	0.4			
12.88	*p*-Menthane-3,8-diol	MTO										0.1	1	0.1
12.97	Citronellol acetate	E							1.6	0.8	1.6	3.5	3.3	3.6
12.98	Citronellyl 2-butenoate	E	0.2											
13.05	Isoeugenol	MTO										0.4		0.4
13.11	Nerol acetate	E	0.1											
13.38	Geraniol acetate	E	3.9	2.4	3.7	0.2	1.8	0.3	6.5	3.3	6.1	4.8	4.5	5
13.44	8-Isopropenyl-1,5-dimethyl-cyclodeca-1,5-diene	STH										0.2	0.2	0.2
13.54	Elemene	STH	0.2			0.4	2	0.5	1.1	0.5	1.1	4.2	3.6	4.3
13.72	Eugenolmethyl	MTO							0.6	0.9	0.6			
13.97	Aromadendrene	STH				0.2	1.4	0.3	1.7	0.7	1.6	0.2	0.2	0.2
13.97	Cis-caryophyllene	STH	1.3	0.8	1.3									
14.11	β-Cubebene	STH										0.1		
14.13	α-Bergamotene	STH	0.1		0.1				0.8	0.4	0.8			
14.37	Trans-isoeugenol	MTO	0.3	0.2	0.2									
14.45	Cis-2-Isopropylbicyclo [4.3.0]non-3-en-8-one	MTO							0.6	0.5	0.6			
14.46	α-Caryophyllene	MTH										0.1	0.1	
14.79	α-Cubebene	STH							1.4	1.2	1.4	1.1	0.7	1.1
14.86	Alpha farnesene	STH							3.8	1.8	3.9			
14.98	Isoeugenol methyl ether	MTO							9.8	10.7	9			
15	7-epi-α-cadinene	STH										0.8	0.7	0.8
15.19	γ-Cadinene	STH							0.3	0.2	0.5	0.6	0.6	0.6
15.24	Cadinene	STH	0.1						0.9	0.4	0.9	2.3	2	2.3
15.25	Cadina-3,9-diene	STH				0.2	1.2	0.3						
15.3	Citronellyl butyrate	E							0.3	0.2	0.4			
15.48	α-cadinene	STH										0.2	0.1	0.1
15.62	β-elemol	MTO	0.3		0.1	3.7	19	5	1.5	1	1.5	7.9	7.4	8
15.68	Geraniol butyrate	E							1.9	1	2			
15.98	Germacrene-4-ol	STO										1.1	1	1.2
16.07	Caryophyllene oxide	STO	1.1	0.6	1	0.2	1.6	0.4	0.3	0.2	0.4			
16.17	Trans-Tricyclo [3.1.0.0(2,4)]hexane, 3,6-diethyl-3,6-dimethyl-,	MTH							0.6	0.3	0.7			
16.39	Lavandulol	MTO										0.2	0.2	0.2
16.4	α-Pinene epoxide	MTO	0.1											
16.56	6-Eudesmen-4-ol	STO				0.4	3.1	0.5						
16.65	γ-Eudesmol	STO				0.3	4.1	0.4	0.3	0.1	0.3	0.6	0.6	0.7
16.78	α-Cadinol	STO										1	1	1
16.92	α-Eudesmol	STO							1	0.6	1	2.2	2	2.2
16.93	10-epi-Elemol	STO	0.1			1	11	1.5						
17.55	Farnesol	STO										0.1	0.1	0.1

Note: MTH: hydrocarbonated monoterpenes, MTO: oxygenated monoterpenes, STH: hydrocarbonated sesquiterpenes, STO: oxygenated sesquiterpenes and E: esters: * The relative abundance is expressed in peak area percentage; the limit consider is ≥0.1% of area in each chromatogram; spectral similarity ≥ 92%. Cells shaded in gray indicate the absence of the corresponding chemical compound in the sample.

**Table 3:** Antibacterial activity of selected pure compounds identified in the analyzed essential oils.

Bacteria Strains		Geraniol %	(+)-(R)-Limonene %	(−)-(R)-Limonene %	Piperitone %
Gram-positive	*S. aureus*	0.63	>5	>5	<0.04
	*E. faecalis*	0.63	>5	>5	ND
Gram-negative	*P. aeruginosa*	0.31	>5	>5	1.25
	*E. coli*	0.63	>5	>5	ND
	*K. pneumoniae*	>5	>5	>5	ND
	*A. baumannii*	0.31	>5	>5	ND
	*E. cloacae*	2.5	>5	>5	ND

MICs expressed as percentage % (*v*/*v*) of the samples tested against the targeted strains; repetitions (*n* = 8); ND means that the results are not determinate.

**Table 4:** Major identified compounds in Cymbopogon essential oils and their respective log P values as indicators of hydrophobicity.

Compound Name	Log P
(Z)-(3,3-Dimethyl)-cyclohexylideneacetaldehyde	2.71
Piperitone	2.85
Geraniol	3.2
Citronellal	3.2
β-Citral	3.45
Camphene	4.22
(+)-2-Carene	4.32
(±)-(R)-Limonene	4.57

## Data Availability

The original contributions presented in this study are included in the article/Supplementary Materials. Further inquiries can be directed to the corresponding authors.

## Supplementary Materials

The following supporting information can be downloaded at https://www.mdpi.com/article/10.3390/antibiotics14050510/s1: Figure S1: Habitat of *Cymbopogon commutatus*; Figure S2: DSL of liposome/the essential oil of *Cymbopogon citratus*; Figure S3: DSL of liposome/the essential oil of *Cymbopogon commutatus*; Figure S4: DSL of liposome/the essential oil of *Cymbopogon nardus*; Figure S5: DSL of liposome/the essential oil of *Cymbopogon winterianus*; Figure S6: GC-MS chromatogram of the essential oil of *Cymbopogon citratus*; Figure S7: GC-MS chromatogram of water-soluble compounds of the essential oil of *Cymbopogon citratus*; Figure S8: GC-MS chromatogram of non-water-soluble compounds of the essential oil of *Cymbopogon citratus*; Figure S9: GC-MS chromatogram of the essential oil of *Cymbopogon commutatus*; Figure S10: GC-MS chromatogram of water-soluble compounds of essential oil of *Cymbopogon commutatus*; Figure S11: GC-MS chromatogram of non-water-soluble compounds of the essential oil of *Cymbopogon commutatus*; Figure S12: GC-MS chromatogram of the essential oil of *Cymbopogon nardus*; Figure S13: GC-MS chromatogram of water-soluble compounds of the essential oil of *Cymbopogon nardus*; Figure S14: GC-MS chromatogram of non-water-soluble compounds of the essential oil of *Cymbopogon nardus*; Figure S15: GC-MS chromatogram of the essential oil of *Cymbopogon winterianus*; Figure S16: GC-MS chromatogram of water-soluble compounds of the essential oil of *Cymbopogon winterianus*; Figure S17: GC-MS chromatogram of non-water-soluble compounds of the essential oil of *Cymbopogon winterianus*; Table S1: Chemical information of the compounds presents in essential oils of *Cymbopogon* species.

## Abbreviations

The following abbreviations are used in this manuscript:

C. citr.	Cymbopogon citratus
C. comm.	Cymbopogon commutatus
C. nard.	Cymbopogon nardus
C. wint.	Cymbopogon winterianus
AMR	Antimicrobial resistance
EO	Essential oil
DLS	Dynamic light scattering
GC-MS	Gas chromatography–mass spectrometry
MIC	Minimum inhibitory concentration
L	Liposome
