# Supplementary material for: Exploring the Antibacterial Potency of Cymbopogon Essential Oils: Liposome Encapsulation and Phytochemical Insights
Source: Antibiotics (Basel). 2025 May 15;14(5):510. doi: 10.3390/antibiotics14050510 (PMC12108311; doi:10.3390/antibiotics14050510)
Supplement: Supplementary file 1 [file antibiotics-14-00510-s001.zip › antibiotics-3590738-supplementary.pdf]

Supporting information

Exploring the Antibacterial Potency of Cymbopogon Essential Oils: Liposome Encapsulation and Phytochemical Insights

Abdirahman Elmi [1,*], Fatouma M. Abdoul-Latif [1], Andréea Pasc [2], Arnaud Risler [2], Stéphanie Philippot [2], Ricardo Gil-Ortiz [3], Dominique Laurain-Mattar [4] and Rosella Spina [4,*]

[1] Centre d'Etudes et de Recherche de Djibouti, Medicinal Research Institute, IRM-CERD, Route de l'Aéroport, Haramous, Djibouti City B.P. 486, Djibouti; fatouma.abdoulatif@cerd.dj (F.M.A.-L.);

[2] Université de Lorraine, CNRS, L2CM, F-54000 Nancy, France; andreea.pasc@univ-lorraine.fr (A.P.); arnaud.risler@univ-lorraine.fr (A.R.); stephanie.philippot@univ-lorraine.fr (S.P.)

[3] Independent Researcher, E-46022 Valencia, Spain; rigilor@alumni.upv.es (R.G.-O.)

[4] Université de Lorraine, INRAE, LAE, F-54000 Nancy, France; dominique.mattar@univ-lorraine.fr

***** Correspondence: abdourahman.elmi@cerd.dj (A.E.); rosella.spina@univ-lorraine.fr (R.S.);

Content

Figure S1. Habitat of *Cymbopogon commutatus*

Figure S2. DSL of liposome/the essential oil of *Cymbopogon citratus*

Figure S3. DSL of liposome/the essential oil of *Cymbopogon commutatus*

Figure S4. DSL of liposome/the essential oil of *Cymbopogon nardus*

Figure S5. DSL of liposome/the essential oil of *Cymbopogon winterianus*

Figure S6. GC-MS chromatogram of the essential oil of *Cymbopogon citratus*

Figure S7. GC-MS chromatogram of water-soluble compounds of the essential oil of *Cymbopogon citratus*

Figure S8. GC-MS chromatogram of non-water soluble compounds of the essential oil of *Cymbopogon citratus*

Figure S9. GC-MS chromatogram of the essential oil of *Cymbopogon commutatus*

Figure S10. GC-MS chromatogram of water-soluble compounds of essential oil of *Cymbopogon commutatus*

Figure S11. GC-MS chromatogram of non-water soluble compounds of the essential oil of *Cymbopogon commutatus*

Figure S12. GC-MS chromatogram of the essential oil of *Cymbopogon nardus*

Figure S13. GC-MS chromatogram of water-soluble compounds of the essential oil of *Cymbopogon nardus*

Figure S14. GC-MS chromatogram of non-water soluble compounds of the essential oil of *Cymbopogon nardus*

Figure S15. GC-MS chromatogram of the essential oil of *Cymbopogon winterianus*

Figure S16. GC-MS chromatogram of water-soluble compounds of the essential oil of *Cymbopogon winterianus*

Figure S17. GC-MS chromatogram of non-water soluble compounds of the essential oil of *Cymbopogon winterianus*

Table S1. Chemical information of the compounds presents in essential oils of Cymbopogon species.

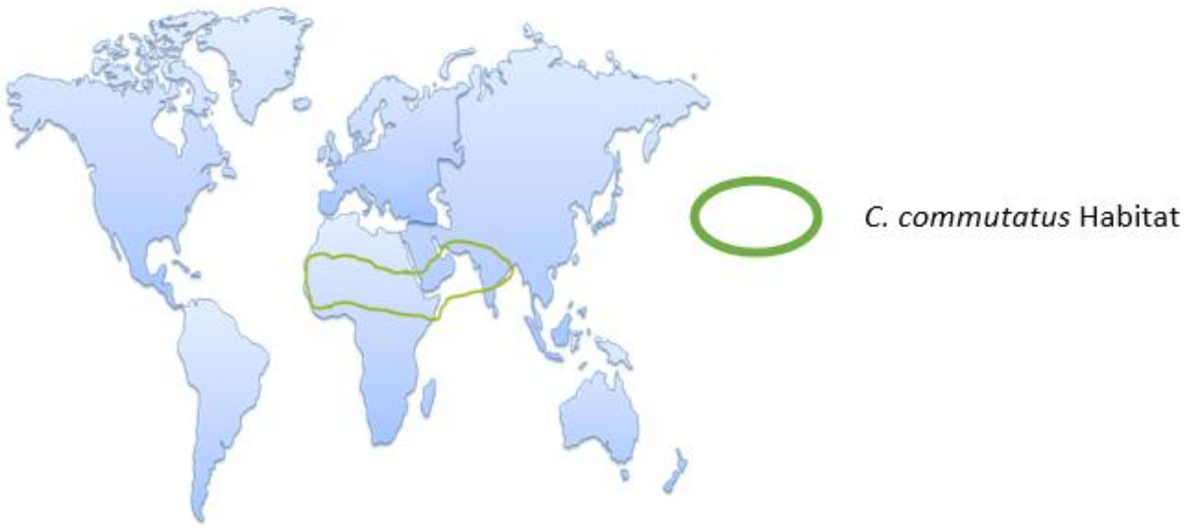

Figure S1: Habitat of *Cymbopogon commutatus, (caws dameer).*

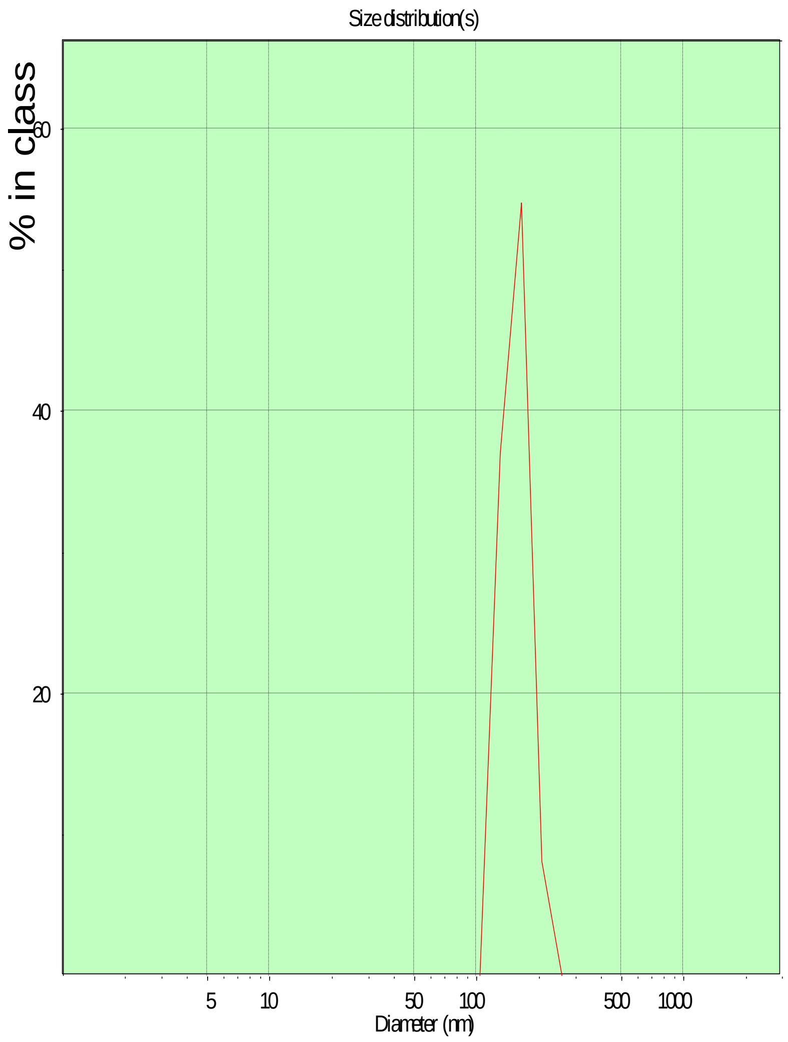

Figure S2. DSL of liposome/the essential oil of *Cymbopogon commutatus*

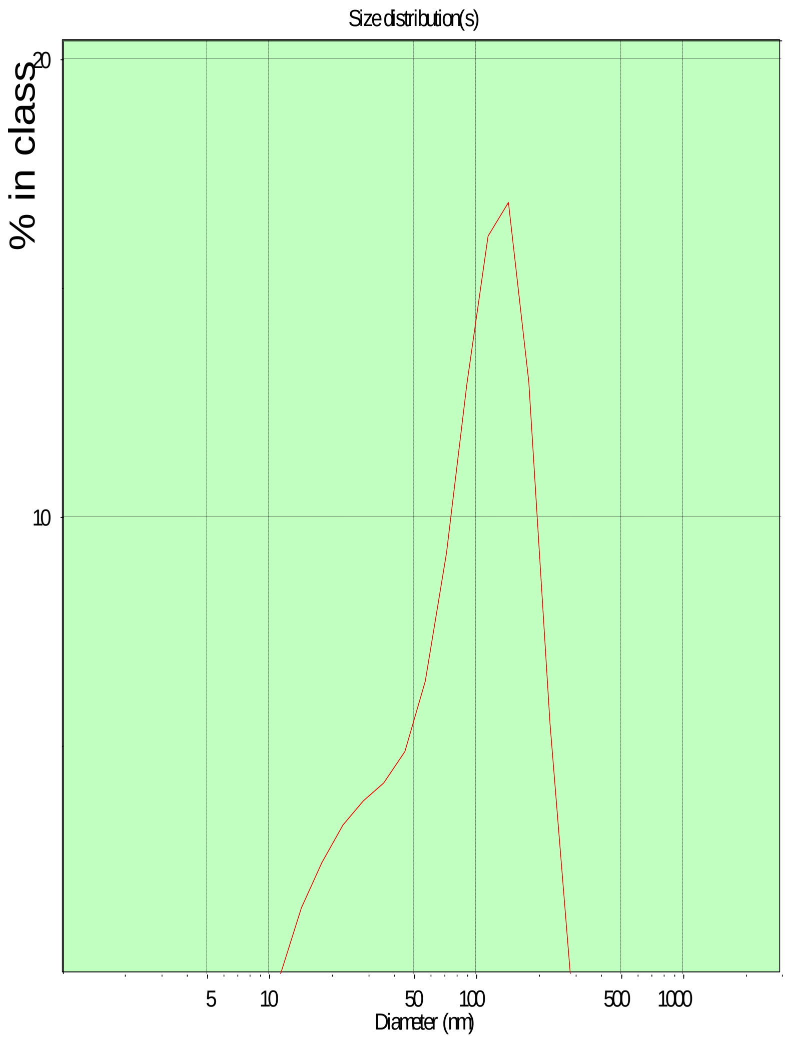

Figure S3. DSL of liposome/the essential oil of *Cymbopogon citratus*

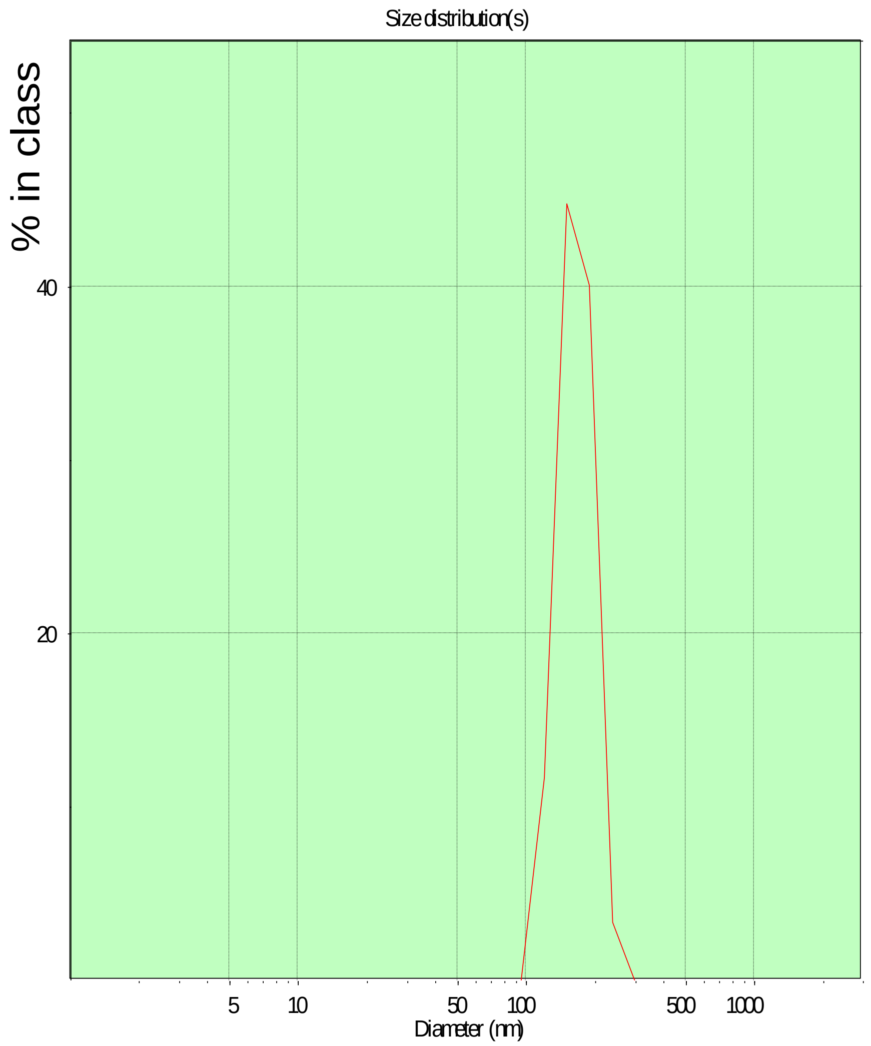

Figure S4. DSL of liposome/the essential oil of *Cymbopogon nardus*

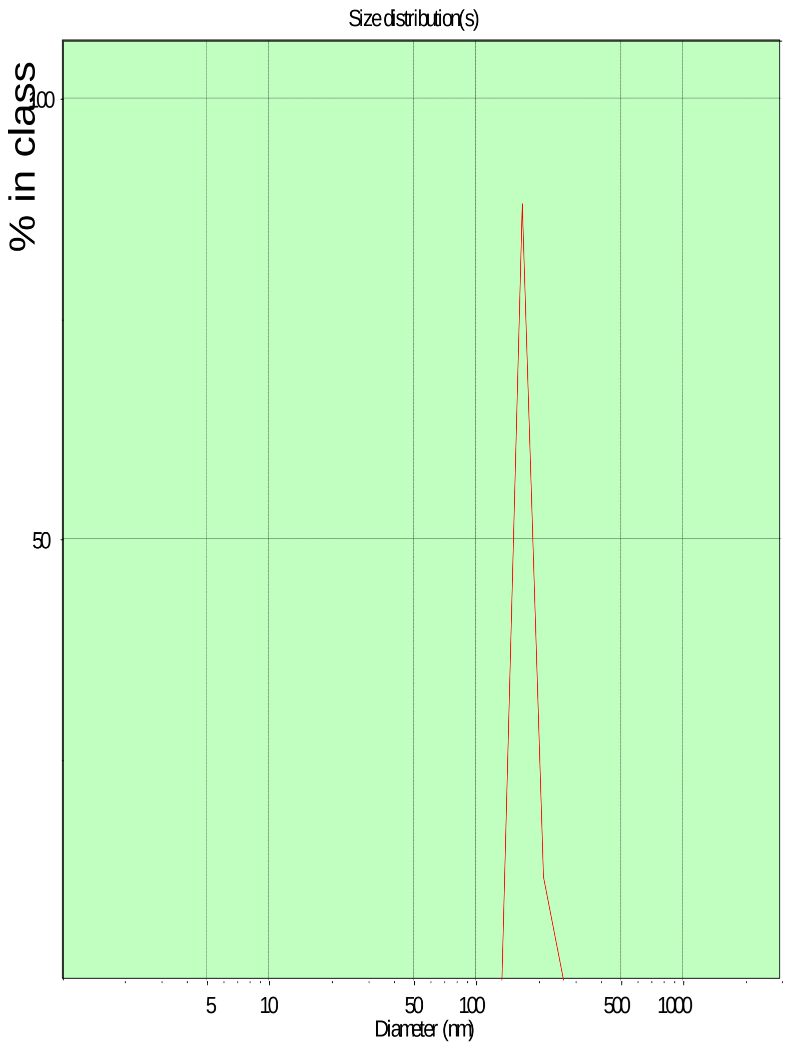

Figure S5. DSL of liposome/the essential oil of *Cymbopogon winterianus*

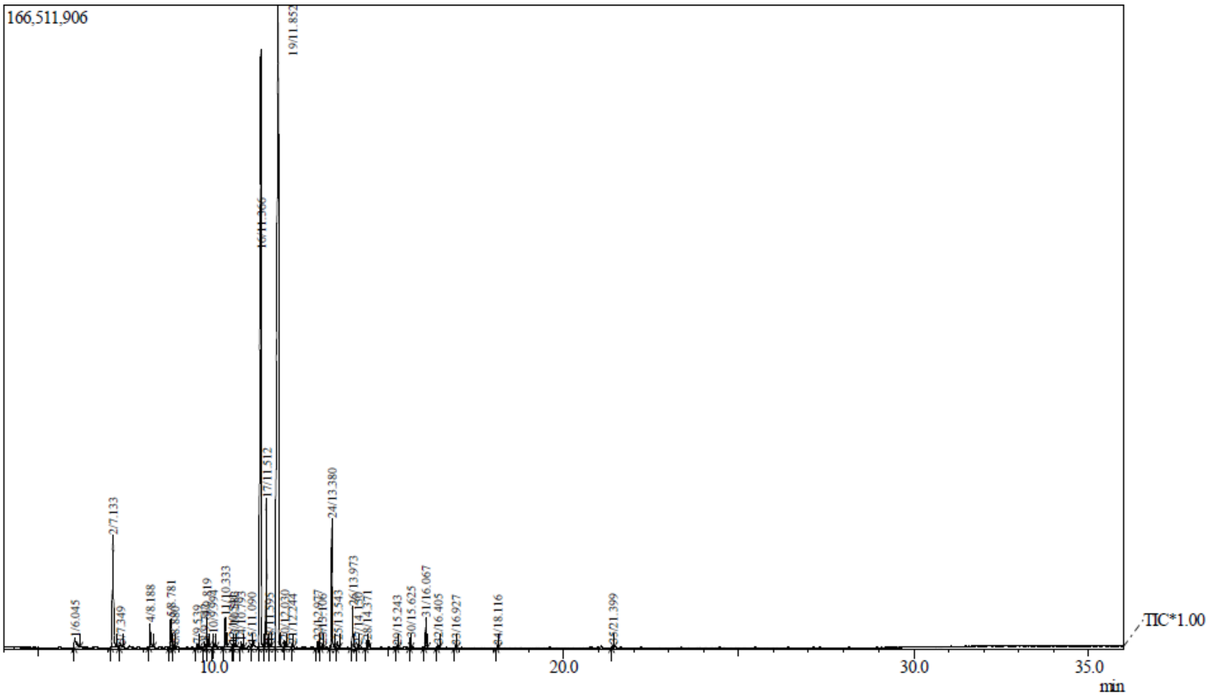

Figure S6. GC-MS chromatogram of the essential oil of *Cymbopogon citratus*

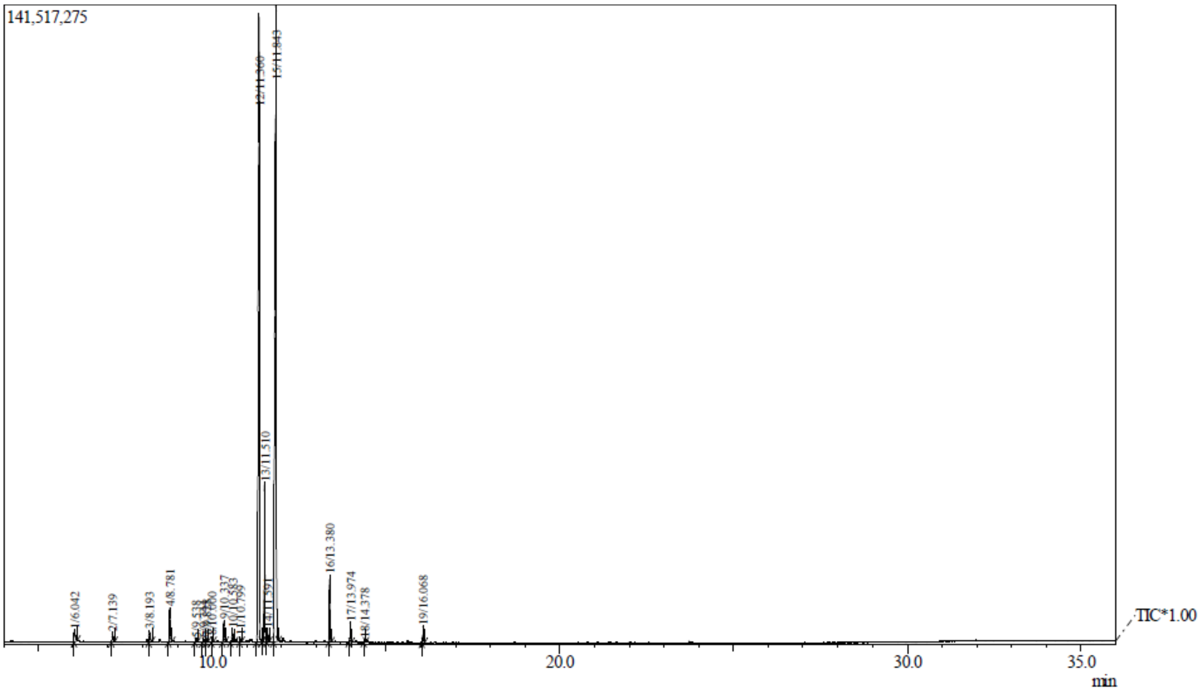

Figure S7. GC-MS chromatogram of water-soluble compounds of the essential oil of *Cymbopogon citratus*

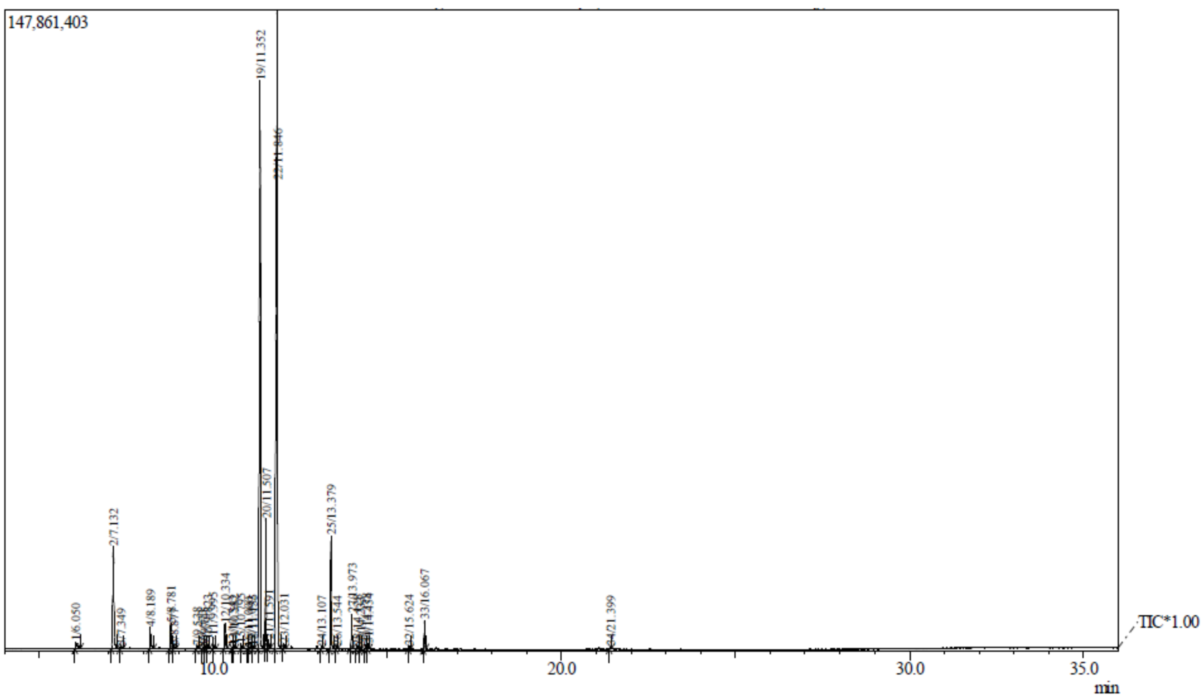

Figure S8. GC-MS chromatogram of non-water soluble compounds of the essential oil of *Cymbopogon citratus*

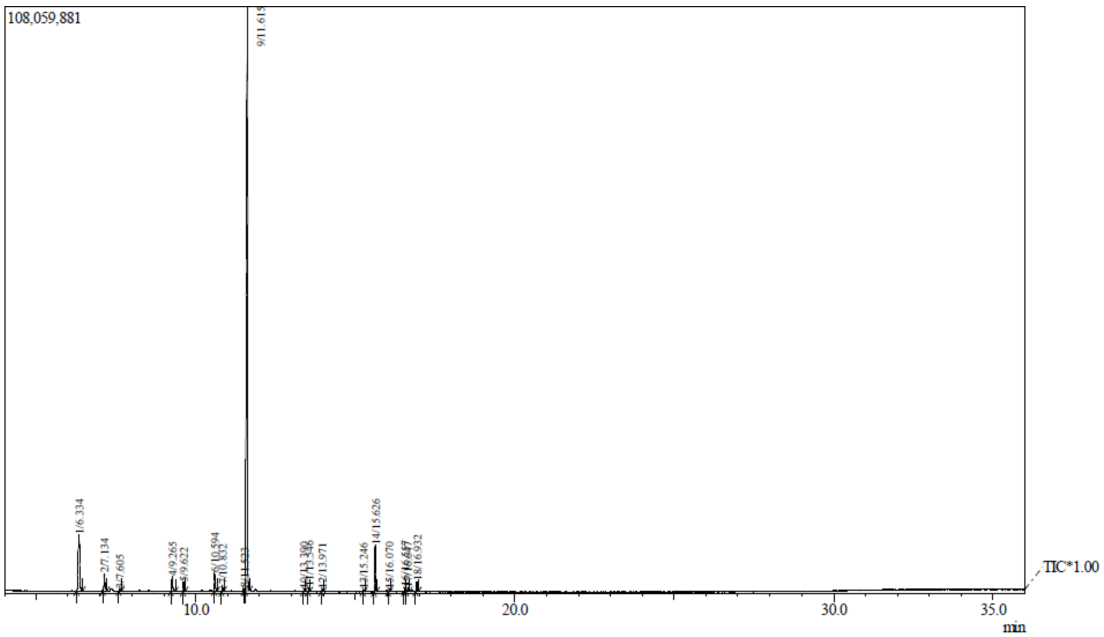

Figure S9. GC-MS chromatogram of the essential oil of *Cymbopogon commutatus*

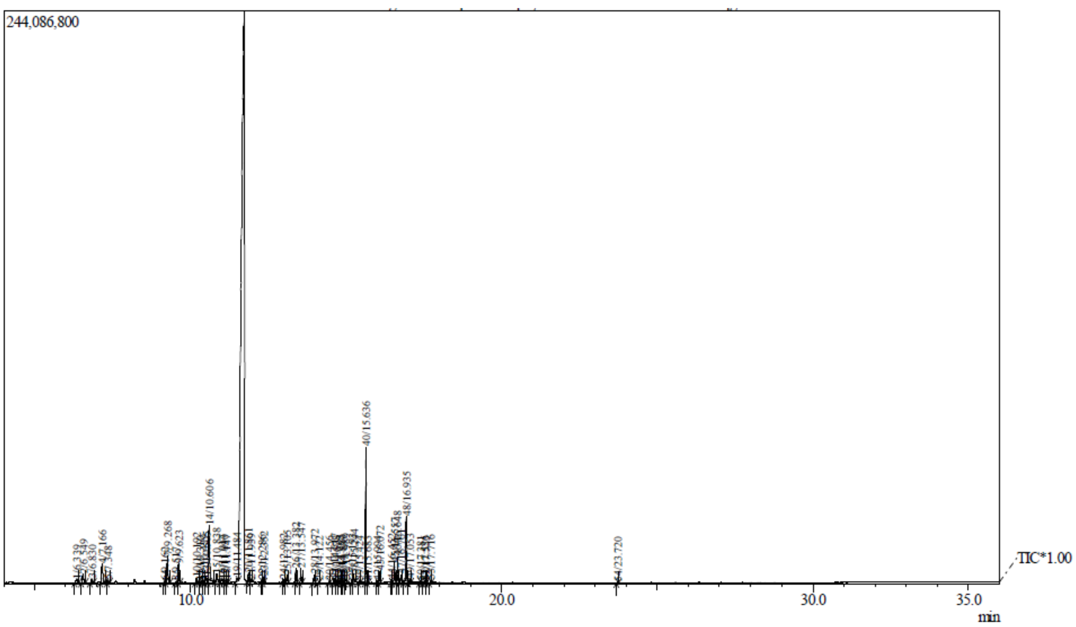

Figure S10. GC-MS chromatogram of water-soluble compounds of essential oil of *Cymbopogon commutatus*

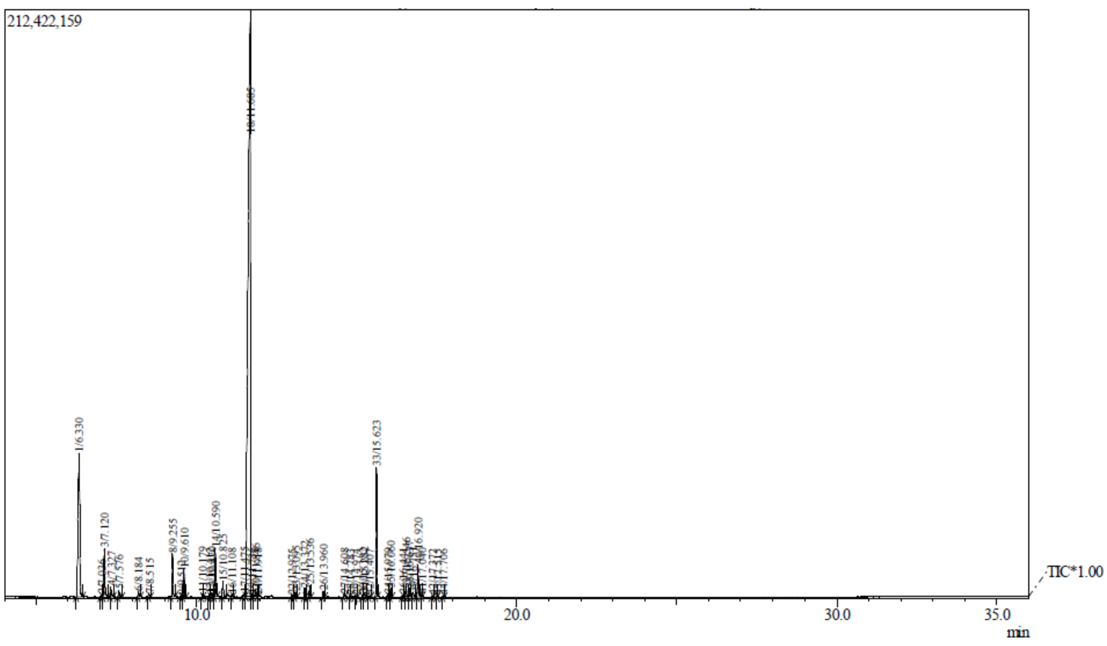

Figure S11. GC-MS chromatogram of non-water soluble compounds of the essential oil of *Cymbopogon commutatus*

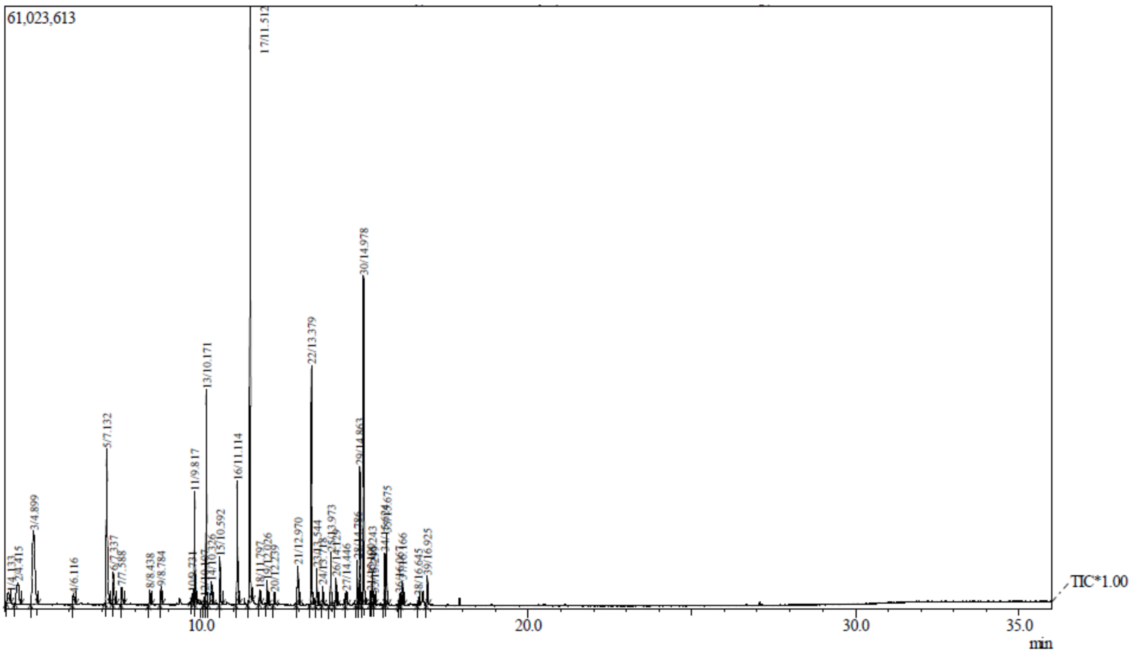

Figure S12. GC-MS chromatogram of the essential oil of *Cymbopogon nardus*

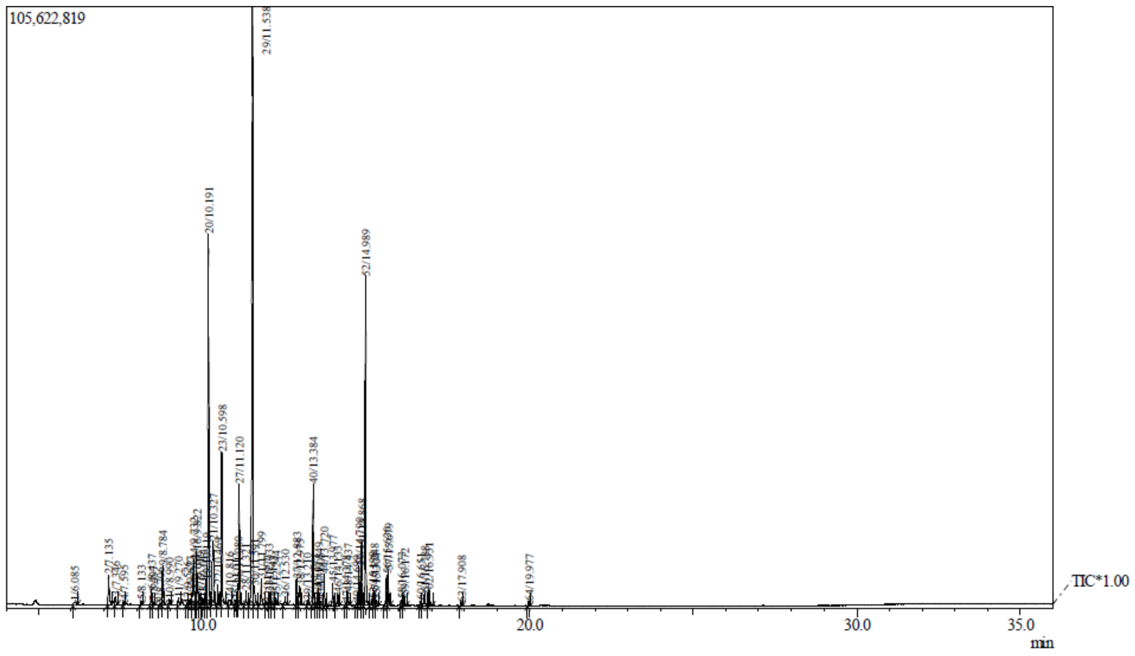

Figure S13. GC-MS chromatogram of water-soluble compounds of the essential oil of *Cymbopogon nardus*

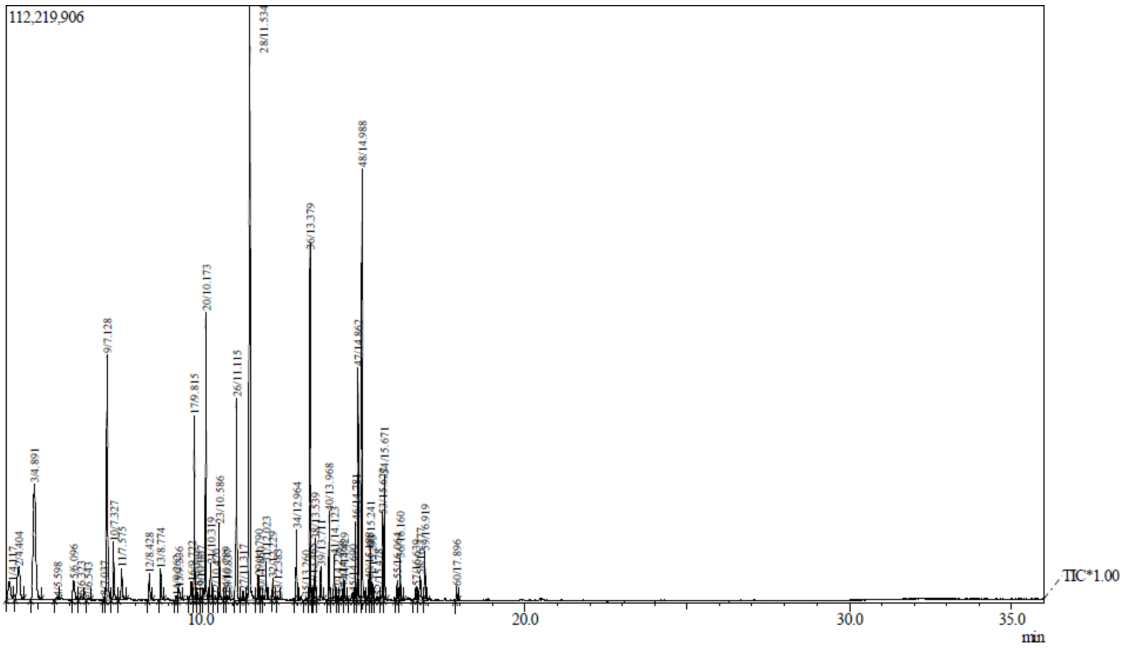

Figure S14. GC-MS chromatogram of non-water soluble compounds of the essential oil of *Cymbopogon nardus*

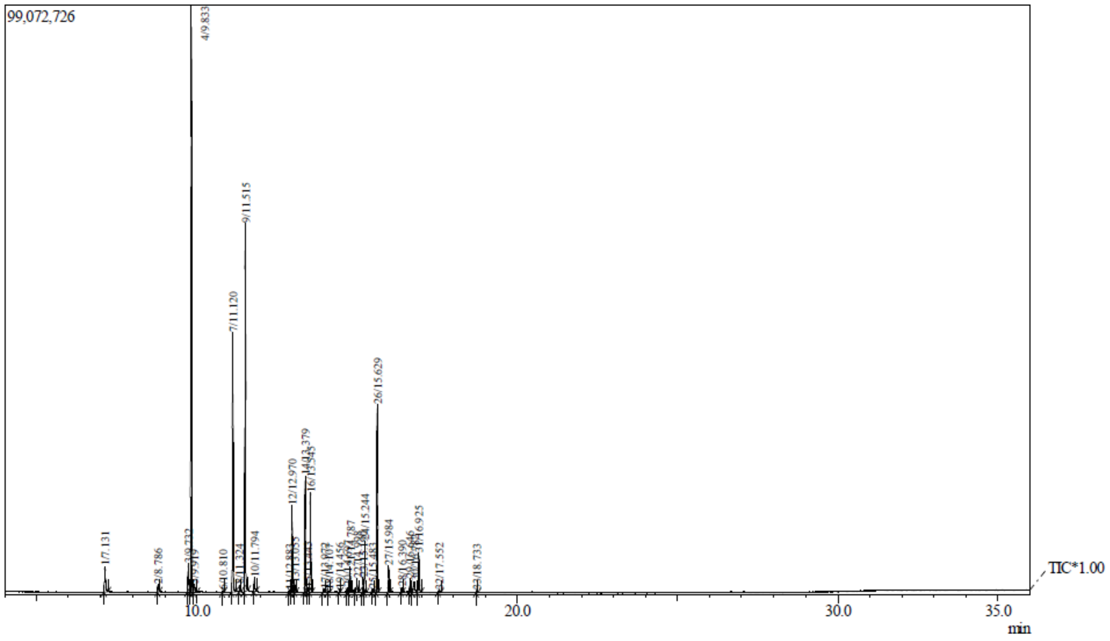

Figure S15. GC-MS chromatogram of the essential oil of *Cymbopogon winterianus*

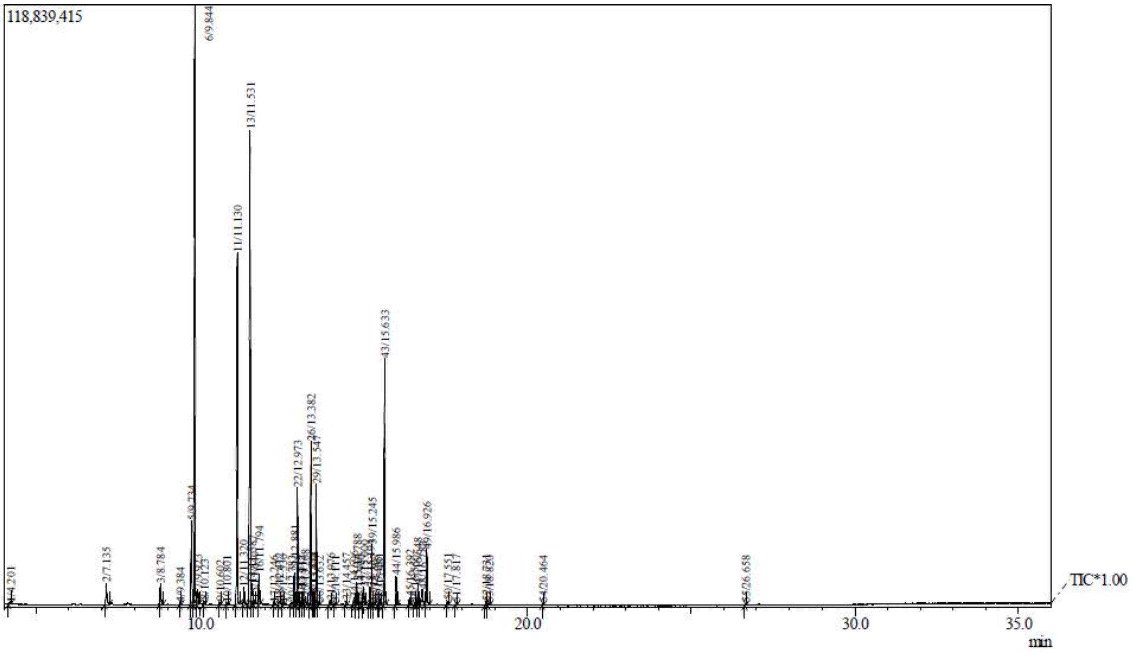

Figure S16. GC-MS chromatogram of water-soluble compounds of the essential oil of *Cymbopogon winterianus*

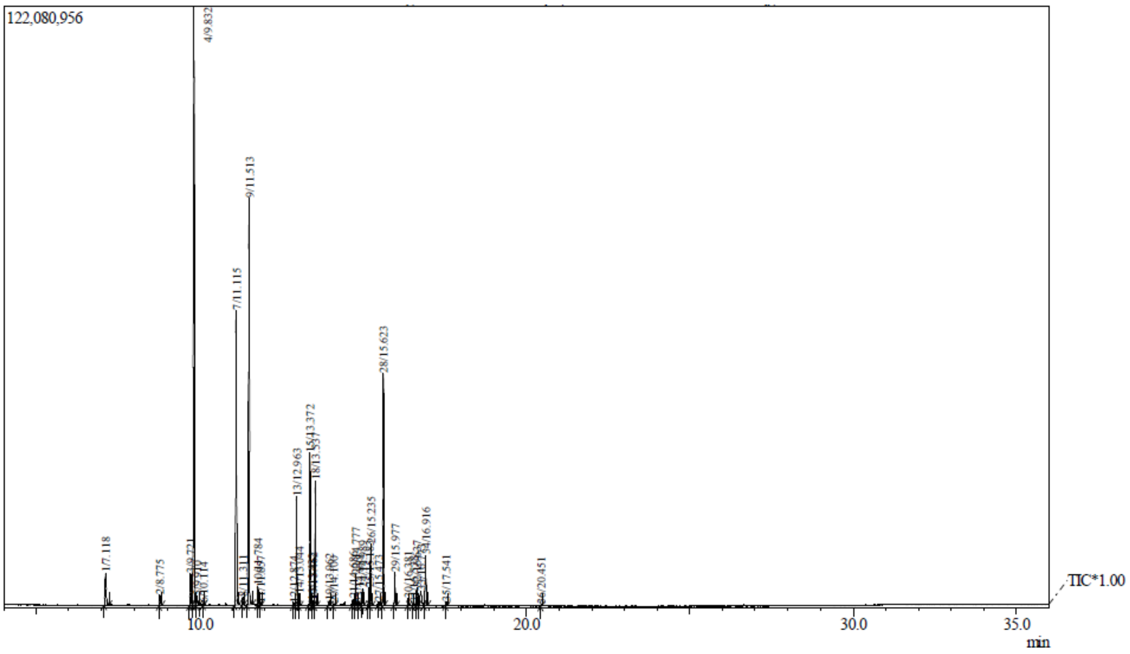

Figure S17. GC-MS chromatogram of non-water soluble compounds of the essential oil of *Cymbopogon winterianus*

Table S1. Chemical information of the compounds presents in essential oils of Cymbopogon species.

RT (min)	Compound name	Chemical formula	Molecular Weight	Retention index
4.13	Tricyclene	C10H16	136	729
4.42	Pinene	C10H16	136	948
4.9	Camphene	C10H16	136	943
6.04	Sulcatone	C8H140	126	938
6.12	β-myrcene	C10H16	136	958
6.33	(+)-2-Carene	C10H16	136	948
7.13	(±)-(R)-Limonene	C10H16	136	1018
7.34	Trans-β-Ocimene	C10H16	136	976
7.59	Ocimene	C10H16	136	976
8.19	4-Nonanone	C9H180	142	1052
8.44	α-Terpinolene	C10H16	136	1052
8.78	β-linalool	C10H180	154	1082
8.88	Myrtanal	C10H160	152	1126
9.26	(E)-p-2-Menthen-1-ol	C10H180	154	1109
9.54	α-Pineneoxide	C10H160	152	961
9.62	(Z)-p-2-Menthen-1-ol	C10H180	154	1109
9.73	p-Menth-8-en-3-ol	C10H180	154	1196
9.74	Trans-4,5-epoxycarene	C10H160	152	948
9.82	Citronellal	C10H180	154	1125
9.92	(-)-Isopulegol	C10H180	154	1196
9.99	1,3,4-trimethyl-3-Cyclohexene-1-carboxaldehyde	C10H160	152	1204
10.11	Camphenol	C10H160	152	1131
10.17	(+)-Borneol	C10H180	154	1138

10.33	Trans-4,5-epoxy-carane	C10H160	152	948
10.33	Terpinen-4-ol	C10H180	154	1137
10.54	(3Z,5Z)-3,5-Octadiene	C8H14	110	832
10.58	Isopinocampheol	C10H180	154	1125
10.59	α-Terpineol	C10H180	154	1143
10.79	Decanal	C10H20O	156	1204
10.81	Palmitaldehyde	C16H32O	240	1800
10.83	Trans-Piperitol	C10H18O	154	1175
11.09	Geranyl nitrile	C10H15N	149	1231
11.11	Citronellol	C10H20O	156	1179
11.32	Neral	C10H16O	152	1174
11.37	β-Citral	C10H16O	152	1174
11.51	Geraniol	C10H18O	154	1228
11.6	Piperitone	C10H16O	152	1158
11.8	α-Citral	C10H16O	152	1174
11.85	(Z)-(3,3-Dimethyl)-cyclohexylideneacetaldehyde	C10H16O	152	1226
12.03	Borneol acetate	C12H20O2	196	1277
12.03	Oxide limonene	C10H16O	152	1031
12.24	Geraniol formate	C11H18O	182	1349
12.88	p-Menthane-3,8-diol	C10H20O2	172	1320
12.97	Citronellol acetate	C12H22O2	198	1302
12.98	Citronellyl 2-butenoate	C14H24O2	224	1509
13.05	Isoeugenol	C10H12O2	164	1410
13.11	Nerol acetate	C12H20O2	196	1352
13.38	Geraniol acetate	C12H20O2	196	1352

13.44	8-Isopropenyl-1,5-dimethyl-cyclodeca-1,5-diene	C15H24	204	1570
13.54	Elemene	C15H24	204	1398
13.72	Eugenolmethyl	C11H14O2	178	1361
13.97	Aromadendrene	C15H24	204	1494
13.97	Cis-caryophyllene	C15H24	204	1494
14.11	β-Cubebene	C15H24	204	1339
14.13	α-Bergamotene	C15H24	204	1430
14.37	Trans-isoeugenol	C10H12O2	164	1410
14.45	Cis-2-Isopropylbicyclo[4.3.0]non-3-en-8-one	C12H18O	178	1331
14.46	α-Caryophyllene	C15H24	204	1579
14.79	α-Cubebene	C15H24	204	1339
14.86	Alpha farnesene	C15H24	204	1458
14.98	Isoeugenol methyl ether	C11H1402	178	1379
15	7-epi-α-cadinene	C15H24	204	1440
15.19	γ-Cadinene	C15H24	204	1435
15.24	Cadinene	C15H24	204	1440
15.25	Cadina-3,9-diene	C15H24	204	1440
15.3	Citronellyl butyrate	C14H26O2	226	1501
15.48	α-cadinene	C15H24	204	1440
15.62	β-elemol	C15H26O	222	1522
15.68	Geraniol butyrate	C14H24O2	224	1550
15.98	Germacrene-4-ol	C15H26O	222	1660
16.07	Caryophyllene oxide	C15H24O	220	1507
16.17	Trans-Tricyclo[3.1.0.0(2,4)] hexane, 3,6-diethyl-3,6-dimethyl-,	C12H20	164	869

16.39	Lavandulol	C10H18O	154	1146
16.4	α-Pinene epoxide	C10H16O	152	961
16.56	6-Eudesmen-4-ol	C15H26O	222	1626
16.65	γ-Eudesmol	C15H260	222	1626
16.78	α-Cadinol	C15H26O	222	1580
16.92	α-Eudesmol	C15H26O	222	1598
16.93	10-epi-Elemol	C15H26O	222	1522
17.55	Farnesol	C15H26O	222	1710
